# NF1-cAMP signaling dissociates cell type–specific contributions of striatal medium spiny neurons to reward valuation and motor control

**DOI:** 10.1371/journal.pbio.3000477

**Published:** 2019-10-10

**Authors:** Laurie P. Sutton, Brian S. Muntean, Olga Ostrovskaya, Stefano Zucca, Maria Dao, Cesare Orlandi, Chenghui Song, Keqiang Xie, Kirill A. Martemyanov

**Affiliations:** Department of Neuroscience, The Scripps Research Institute, Jupiter, Florida, United States of America; ICM - Institut du Cerveau et de la Moelle épinière Hôpital Pitié-Salpêtrière 47, bd de l'Hôpital, FRANCE

## Abstract

The striatum plays a fundamental role in motor learning and reward-related behaviors that are synergistically shaped by populations of D1 dopamine receptor (D1R)- and D2 dopamine receptor (D2R)-expressing medium spiny neurons (MSNs). How various neurotransmitter inputs converging on common intracellular pathways are parsed out to regulate distinct behavioral outcomes in a neuron-specific manner is poorly understood. Here, we reveal that distinct contributions of D1R-MSNs and D2R-MSNs towards reward and motor behaviors are delineated by the multifaceted signaling protein neurofibromin 1 (NF1). Using genetic mouse models, we show that NF1 in D1R-MSN modulates opioid reward, whereas loss of NF1 in D2R-MSNs delays motor learning by impeding the formation and consolidation of repetitive motor sequences. We found that motor learning deficits upon NF1 loss were associated with the disruption in dopamine signaling to cAMP in D2R-MSN. Restoration of cAMP levels pharmacologically or chemogenetically rescued the motor learning deficits seen upon NF1 loss in D2R-MSN. Our findings illustrate that multiplex signaling capabilities of MSNs are deployed at the level of intracellular pathways to achieve cell-specific control over behavioral outcomes.

## Abstract 2

A mouse genetic study reveals that the multifaceted signaling protein neurofibromin (known for its role in the human genetic disease neurofibromatosis type 1) plays a key role in differential routing of motor and reward signals in populations of striatal medium spiny neurons.

## Introduction

The striatum is the main input nucleus of the basal ganglia system, a subcortical structure critically involved in both motor control and motivational processes [1]. Much of the information processing in the striatum is performed by two populations of GABAergic medium spiny neurons (MSNs) that have divergent efferent targets, distinct neuromodulatory features, and influence behavior in an opponent manner [2,3]. Most notably, MSN populations are delineated by the selective expression of either dopamine receptor D1R (D1R-MSN) or D2R (D2R-MSN), activation of which by dopamine triggers a stimulatory or inhibitory response, respectively [4,5]. MSNs located in the dorsal striatum that encompasses the caudate/putamen region generally receive dopaminergic innervation from the substantia nigra pars compacta (SNc) and project to either the substantia nigra pars reticulata (D1R-MSN) or to the external segment of the globus pallidus (D2R-MSN). In contrast, MSNs of the ventral striatum comprising the nucleus accumbens (NAc) receive dopaminergic inputs from the neurons in the ventral tegmental area and in return project to neurons in the ventral pallidus [6]. It is generally thought that MSNs in the dorsal striatum play a greater role in motor control, whereas neurons of the NAc are involved more in motivational control, reward, and incentive learning [7,8]. However, there is ample evidence indicating that both regions contribute to various aspects and phases of striatal-controlled behaviors [9,10].

The classic model of striatal involvement in motor control posits that the two efferent pathways exert opposing influence, whereby activation of the D1R-MSNs increases movements and promotes action initiation, while activation of the D2R-MSNs inhibits locomotion and suppresses execution of motor tasks [11,12]. Similarly, opposing contributions of D1R-MSNs and D2R-MSNs have been shown to shape motivated behavior and incentive learning. Activation of D1R-MSNs is sufficient to drive reward-related behaviors, whereas inactivation suppresses these responses [13,14]. In contrast, inhibition of the D2R-MSN pathway enhances drug-induced sensitization and motivation behaviors, whereas activation diminishes these effects [13,15,16]. Recently, this model has been revised to accommodate for complementary and convergent contributions of D1R-MSNs and D2R-MSNs in initiation of motor action [17–19] and responses to addictive drugs [20–22]. This suggest that changes within the MSN signaling cascades likely program individual behavioral responses; yet our understanding of molecular and cellular mechanisms that delineate the impact of striatal neurons on diversity of behavioral outcomes is limited.

Responses of MSNs are tightly controlled by several neuromodulators, including acetylcholine, glutamate, adenosine, and opioids, that play critical roles in regulating striatal-mediated behaviors. Much of these effects is mediated by the G protein coupled receptors (GPCRs) that act by engaging intracellular second messenger pathways to influence key MSN properties, including excitability, synaptic plasticity, and spine morphology. These GPCRs converge on two key pathways: cAMP and Ras-regulated kinases. Both pathways have an extensive documented role in controlling D1R-MSN and D2R-MSN responses and striatal-mediated behaviors. For example, changes in striatal cAMP signaling are thought to drive long-term adaptive changes in ion channel function and transcription, leading to drug-reinforcement and dependence [23–25]. Similarly, targeting of molecules involved in cAMP biosynthesis or its downstream effectors severely impacts motor learning and reward-related behaviors [17,26,27]. Deletion of kinases regulated by Ras such as extracellular signal regulated kinase (ERK), glycogen synthase kinase (GSK-3), and mammalian target of rapamycin (mTOR) in discrete populations of MSNs also affects motor activity and/or responses to psychostimulants [28–30], and similar effects are observed upon changes in Ras activity [31,32]. Thus, by engaging different intracellular cascades, neuromodulatory GPCRs in the striatum are able to project a powerful influence on MSN physiology to control diverse behavioral responses. One intriguing possibility provided by these observations is that neuromodulatory processing in MSNs is multiplexed at the molecular level, where several channels are intertwined to modulate discrete behavioral outputs. Testing this model would require dissociating contributions of individual signaling cascades to controlling reward-related and motor outcomes in an MSN population–specific manner.

Neurofibromin 1 (NF1) is a large multidomain signaling molecule with the potential to provide clues into the organization of striatal neuromodulatory mechanisms. Mutations in the NF1 gene cause Neurofibromatosis type 1 a genetic disorder characterized by multiple benign and malignant tumors with prominent neuropsychiatric symptoms including learning and attention deficits, as well as motor impairments [33]. NF1 functions as a Ras-specific GTPase activating protein (RasGAP), and this property has been implicated in memory consolidation [34–37]. In addition, NF1 also acts as a positive regulator of cAMP levels by mediating GPCR-dependent activation of adenylate cyclase [37–39]. The mechanism by which NF1 regulates cAMP remains controversial, and both Ras-dependent as well as Ras-independent modes have been suggested [39–41]. Curiously, NF1 has been linked as a direct effector of GPCR signaling in striatal neurons, with a profound impact on reward and reinforcing behaviors [31]. Furthermore, some neuropsychiatric features in NF1 patients and animal models also suggest the involvement of striatal circuitry [42,43]. Thus, NF1 presents a critical signaling hub in routing neuromodulatory GPCR signals, offering a convenient model to understand multiplexing mechanisms in regulating downstream effector pathways and their impact on striatal-mediated behavior.

Here, we demonstrate that intracellular signaling pathways processing neurotransmitter inputs onto MSNs can be dissociated in a cell-specific manner, with distinct contribution to motor learning and reward-related behaviors. We report that a key role in routing neuromodulatory GPCR signals to diverging behavioral outcomes belongs to NF1, which molecularly dissociates contributions of MSN populations to behavior, and dissect the mechanism that enables this action.

## Results

### Selective contributions of D1R-MSN signaling to opioid reward

**Fig 1:**
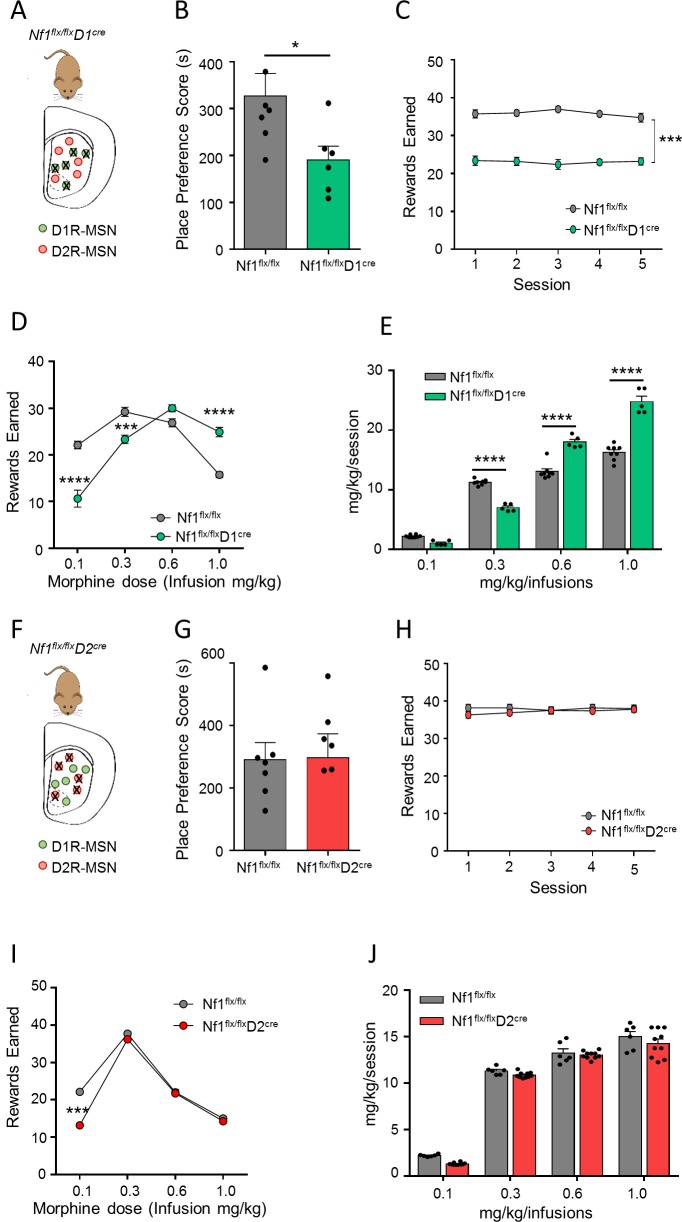
Lack of NF1 in D1R-MSNs but not in D2R-MSNs reduces opioid reward. Scheme of NF1 deletion from (A) D1R-MSNs or (F) D2R-MSNs (mouse graphic was adapted from [44]). Effects of morphine-induced CPP (10 mg/kg) for (B) *Nf1*^*flx/flx*^*D1*^*Cre*^ mice (*n* = 6–7 mice/group) or (G) *Nf1*^*flx/flx*^*D2*^*Cre*^ mice (*n* = 7 mice/group). Place preference scores are calculated as the difference between time spent in the drug-paired side during post-conditioning versus preconditioning tests. *P < 0.05, Student *t* test. Rewards earned during morphine self-administration at 0.3 mg/kg/infusion for (C) *Nf1*^*flx/flx*^*D1*^*Cre*^ (*n* = 5–8 mice/group) and (H) *Nf1*^*flx/flx*^*D2*^*Cre*^ mice (*n* = 6–10 mice/group). ***P < 0.001, two-way RM ANOVA. Self-administration criteria were set under a fixed-ratio 5 with a time-out 20-second schedule of reinforcement. Number of infusions earned during morphine self-administration at varying doses (mg/kg/infusions) for (D) *Nf1*^*flx/flx*^*D1*^*Cre*^ mice and (I) *Nf1*^*flx/flx*^*D2*^*Cre*^ mice. ***P < 0.001, ****P < 0.0001 compared with *Nf1*^*flx/flx*^ mice, two-way RM ANOVA. Intake of morphine self-administration calculated from the last three stable sessions for (E) *Nf1*^*flx/flx*^*D1*^*Cre*^ mice and (J) *Nf1*^*flx/flx*^*D2*^*Cre*^ mice. ****P < 0.0001, two-way RM ANOVA. Data are represented as mean ± SEM. Underlying data for this figure can be found in S1 Data. CPP, conditioned place preference; D1R, D1 dopamine receptor; D2R, D2 dopamine receptor; MSN, medium spiny neuron; NF1, neurofibromin 1; RM-ANOVA, repeated measures analysis of variance.

We began testing the idea that intracellular signaling may be differentially routed in a cell type–specific manner to dissociate striatal-mediated behaviors by taking advantage of a mouse model with the loss of NF1 for its key impact on striatal signaling [31]. To assess contributions of individual MSN populations to behavior controlled by NF1, we generated mice with a deletion of NF1 in either D1R- or D2R-expressing neurons by crossing *Nf1*^*flx/flx*^ mice with bacterial artificial chromosome (BAC) transgenic mice harboring a hemizygous allele for Cre recombinase under the D1 or the D2 promoter (Fig 1A, Fig 1F, S1A Fig and S1C Fig). The resulting *Nf1*^*flx/flx*^*D1*^*cre*^ mice and the wild-type *Nf1*^*flx/flx*^ mice were first evaluated in a conditioned place preference (CPP) paradigm in which reward was assessed by the preference for a drug-paired environment. We found that at an intermediate dose of morphine (10 mg/kg), *Nf1*^*flx/flx*^*D1*^*cre*^ mice spent significantly less time in the drug-paired chamber compared with their wild-type counterparts, *Nf1*^*flx/flx*^ mice, suggesting that the deletion of NF1 in D1R-MSNs diminishes morphine reward (Fig 1B). To confirm this finding, we utilized a self-administration paradigm to assess reinforcing effects of drugs directly. Initially, mice were trained on a two-lever operant task to respond to a food reward to establish the motor skill of lever pressing. During food self-administration, both *Nf1*^*flx/flx*^ and *Nf1*^*flx/flx*^*D1*^*cre*^ mice learned to earn food rewards; however, *Nf1*^*flx/flx*^*D1*^*cre*^ mice acquired set criteria earlier than control *Nf1*^*flx/flx*^ mice (S1B Fig). Following food training, mice were implanted with intravenous jugular catheters and tested for lever-pressing behavior resulting in morphine infusions. Consistent with observations in the place preference test, *Nf1*^*flx/flx*^*D1*^*cre*^ mice exhibited fewer active lever presses when self-administering an intermediate dose of 0.3 mg/kg per infusion (Fig 1C). Analysis of the dose-response relationship revealed a prominent rightward shift, indicating that *Nf1*^*flx/flx*^*D1*^*cre*^ mice needed a higher dose of morphine than *Nf1*^*flx/flx*^ mice to achieve similar reinforcement levels (Fig 1D). Similarly, the total morphine intake per session at 0.3 mg/kg is also reduced in *Nf1*^*flx/flx*^*D1*^*cre*^ mice. However, at higher doses (0.6 and 1 mg/kg/infusion), *Nf1*^*flx/flx*^*D1*^*cre*^ mice had significantly greater total intake of morphine relative to controls (Fig 1E). This rightward shift on the dose-response curve is further evident when the total morphine intake per session is calculated (Fig 1E) and is reflective of the reduced sensitivity of *Nf1*^*flx/flx*^*D1*^*cre*^ mice for the rewarding effects of morphine.

Next, we evaluated mice with a deletion of NF1 in D2R-MSNs (S1C Fig) in the same panel of behavioral tests. When tested in the CPP paradigm, *Nf1*^*flx/flx*^*D2*^*cre*^ mice developed a robust place preference for morphine, with place preference scores similar to their control littermates (Fig 1G). In the self-administration paradigm, *Nf1*^*flx/flx*^*D2*^*cre*^ mice learned food self-administration to the set criteria; however, animals took significantly longer to establish this behavior compared with *Nf1*^*flx/flx*^ controls (S1D Fig). Following implantation of intravenous jugular catheters and switching to morphine, we found that *Nf1*^*flx/flx*^*D2*^*cre*^ mice had to relearn lever pressing, and as such, we assessed self-administration when this behavior was stabilized. Once this task was acquired, *Nf1*^*flx/flx*^*D2*^*cre*^ readily self-administered morphine at a dose of 0.3 mg/kg infusions with no difference from the control *Nf1*^*flx/flx*^ littermates (Fig 1H). Analysis of the dose-response relationship showed both genotypes earned similar rewards at intermediate to high doses of 0.3, 0.6, or 1.0 mg/kg, but *Nf1*^*flx/flx*^*D2*^*cre*^ mice engaged in fewer lever presses at the low dose of 0.1 mg/kg, likely due to general difficulties with task acquisition, exacerbated by a low reinforcing regimen (Fig 1I). Nevertheless, both genotypes responded similarly with increased total morphine intake across all doses used (Fig 1J). Collectively, these data indicate that sensitivity to the rewarding properties of morphine is not influenced by eliminating NF1 in D2R-MSNs.

### Common mechanisms controlling intrinsic excitability in both populations of striatal MSNs

Changes in the intrinsic properties of MSNs have been shown to shape the rewarding and reinforcing properties of opioid use [45,46]. Thus, to begin probing mechanisms underlying cell-selective effects revealed by NF1 loss, we examined changes in the biophysical properties of MSNs by whole cell patch clamp recordings. First, we examined the consequences of pan-striatal NF1 elimination without distinguishing between MSN populations by crossing *Nf1*^*flx/flx*^ mice with the *Rgs9*^*Cre*^ driver line, which expresses Cre recombinase in all striatal neurons. In drug-naïve mice, we found no difference between *Nf1*^*flx/flx*^*Rgs9*^*cre*^ and *Nf1*^flx/flx^ mice in several measures of intrinsic properties of MSNs in the NAc (S2 Fig). In the basal state, NAc neurons in *Nf1*^*flx/flx*^*Rgs9*^*cre*^ and *Nf1*^*flx/flx*^ mice exhibited similar resting membrane potentials (RMPs), input resistance, and firing patterns as their control littermates (S2A–S2E Fig). Next, we compared neuronal properties in both genotypes after repeated morphine administration (twice daily for 5 consecutive days, 10 mg/kg) that emulated the drug exposure schedule of the rewarding effects of morphine in the behavioral tasks. Following the last morphine injection, intrinsic excitability of NAc neurons in *Nf1*^*flx/flx*^ mice was significantly diminished compared with vehicle treated *Nf1*^*flx/flx*^ mice, as evident from the reduction in the number of action potentials (APs) generated in response to current injections (S2A Fig and S2B Fig) and elevation in firing threshold (S2C Fig). In contrast, morphine failed to change the intrinsic excitability of NAc neurons in *Nf1*^*flx/flx*^*Rgs9*^*cre*^ mice (S2A Fig and S2B Fig). Although there was no difference in the RMP across genotypes and treatments (S2D Fig), morphine selectively decreased input resistance in *Nf1*^*flx/flx*^ MSNs and not in *Nf1*^*flx/flx*^*Rgs9*^*cre*^ neurons (S2E Fig), suggesting that NF1 may be required for opioids to engage inhibitory ion channels. These changes in excitability were selective, as we did not observe an impact of NF1 on either presynaptic plasticity exemplified by unchanged paired pulse facilitation or postsynaptic plasticity reflected by unaltered α-amino-3-hydroxy-5-methyl-4-isoxazolepropionic acid receptor (AMPAR)/N-methyl-D-aspartate receptor (NMDAR) ratio under both basal drug-naïve conditions and following repeated morphine treatment (S2F–S2I Fig). Overall, these data show that MSNs lacking NF1 are resistant to morphine-induced reduction in excitability and suggest that Mu opioid receptor (MOR) signaling via NF1 plays an essential role in dictating response to morphine.

Considering that our observations show the role of NF1 in controlling morphine effects in the striatum, we explored a reciprocal regulation testing the possibility that morphine may provide feedback by regulating NF1 in turn. Indeed, we found that morphine injections (10 or 20 mg/kg) caused marked up-regulation of NF1 protein levels in the NAc (S3 Fig). This effect was specific for NF1, as we did not observe any significant changes in levels of other prominent Ras regulators, including p120GAP, SynGAP, Sos1, RasGRP1, and RasGRF2. These observations further reinforce the connection between opioid effects and NF1 and suggest its involvement in adaptive changes in striatal neurons.

**Fig 2:**
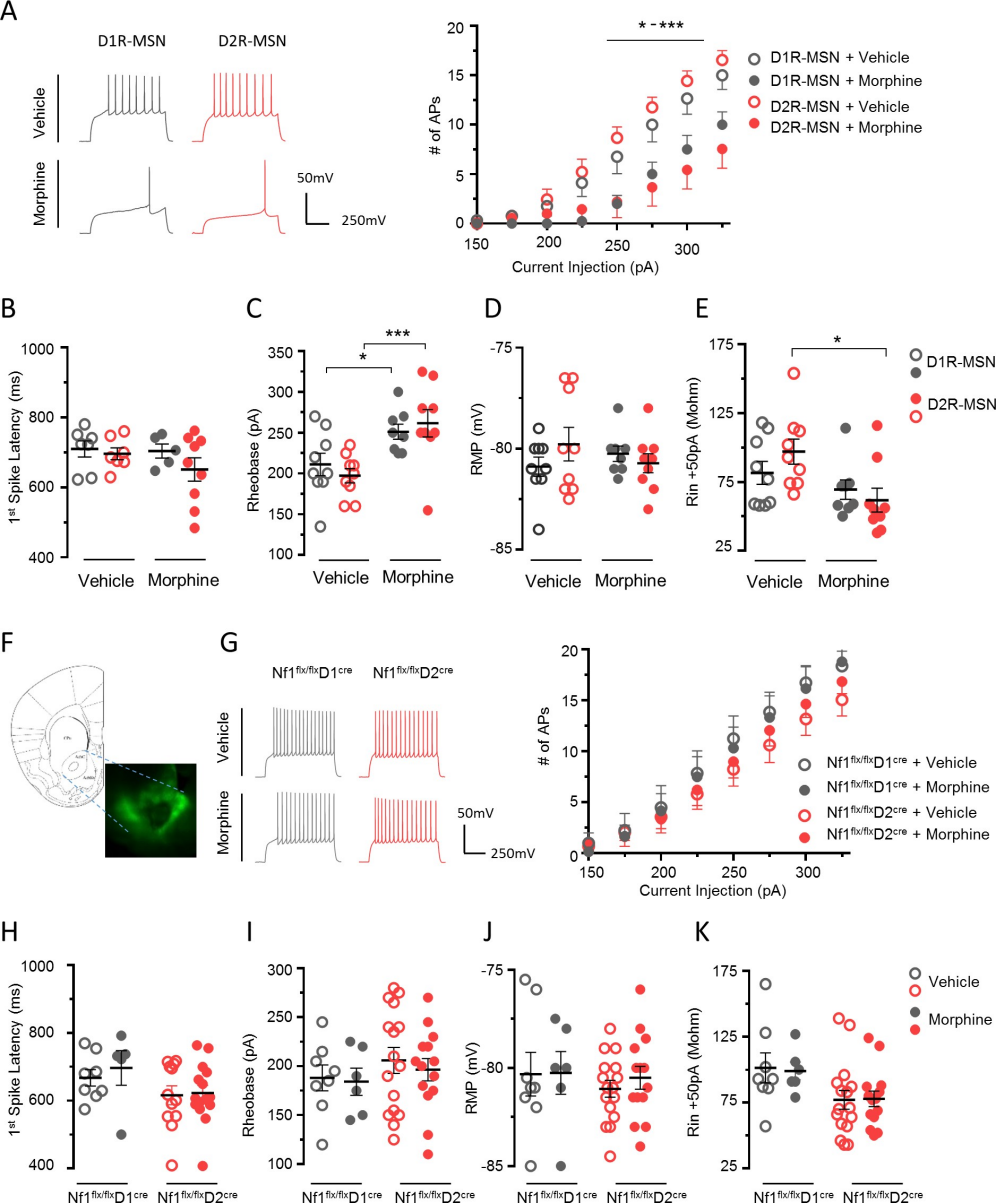
MSNs lacking NF1 are resistant to morphine-induced reduction in excitability. (A) Representative traces at 250-pA injection level and the mean number of APs generated for a given level of current injection in D1R-MSNs and D2R-MSNs following morphine administration. *n* = 8–9 neurons/group. Comparison of the (B) first spike latency (ms) (C) firing threshold (rheobase), (D) RMP, and (E) input resistance (Rin) in D1R-MSNs and D2R-MSNs of drug-naïve and morphine-treated mice. *n* = 8–9 neurons/group. (F) Image of the NAc in *Nf1*^*flx/flx*^*D2*^*Cre*^ mouse stereotaxically injected with AAV9-Flex-EGFP. (G) Representative traces and the mean number of APs generated for a given level of current injection in *Nf1*^*flx/flx*^*D1*^*Cre*^ and *Nf1*^*flx/flx*^*D2*^*Cre*^ mice following morphine administration. *n* = 8–16 neurons/group. Comparison of the (H) first spike latency (ms), (I) firing threshold (rheobase), (J) RMP, and (K) input resistance (Rin). *n* = 5–16 neurons/group. *P < 0.05, ***P < 0.001, two-way RM ANOVA. Data are represented as mean ± SEM. Underlying data for this figure can be found in S1 Data. AP, action potential; D1R, D1 dopamine receptor; D2R, D2 dopamine receptor; MSN, medium spiny neuron; NAc, nucleus accumbens; NF1, neurofibromin 1; RM-ANOVA, repeated measures analysis of variance; RMP, resting membrane potential.

We next assessed cell specificity of NF1’s involvement in mediating the effects of morphine administration on MSN electrophysiological properties. For this, we first recorded from MSNs in NAc of Drd2–green fluorescent protein (GFP) reporter mice following chronic morphine injections. D2R-MSNs were identified as GFP-positive cells, whereas non-GFP neurons were considered to be D1R-MSNs, even though an occasional misidentification cannot be completely ruled out. We found that both MSN populations exhibited a decrease in excitability in response to morphine (Fig 2A), with no difference in latency to the first AP in response to rheobase current injection (Fig 2B). Morphine increased the rheobase in both MSN populations (Fig 2C), whereas a decrease in input resistance was only found in D2R-MSN (Fig 2E). RMP was unaffected by morphine treatment (Fig 2D). To examine cell specificity of morphine-induced adaptations, we established MSN identity by stereotaxic delivery of AAV9-Flex-eGFP into the NAc of either *Nf1*^*flx/flx*^*D1*^*Cre*^ or *Nf1*^*flx/flx*^*D2*^*Cre*^ mice (Fig 2F). Excitability measurements revealed that both D1R-MSNs and D2R-MSNs lacking NF1 were resistant to morphine, as their excitability did not differ from that of vehicle treated mice (Fig 2G). This result is in contrast to wild-type mice, in which morphine decreased excitability in both MSN populations. Rheobase, RMP, and input resistance did not differ between saline and morphine groups in either D1R-MSNs and D2R-MSNs of mice lacking NF1 (Fig 2H–2K). Overall, these data suggest that NF1 is critically involved in morphine-induced modulation of excitability in both populations of striatal MSNs.

Because the D2^cre^ driver line is also active in cholinergic interneurons (CINs), we sought to further test the effects of Nf1 ablation in this neuronal population. We found no differences in the intrinsic excitability, RMP, and input resistance of CINs between *Nf1*^*flx/flx*^*D2*^*Cre*^ and *Nf1*^*flx/flx*^ mice (S4 Fig), suggesting that NF1 may not be involved in regulating basal activity of these neurons.

### Selective contributions of D2R-MSNs and NF1 to motor learning

**Fig 3:**
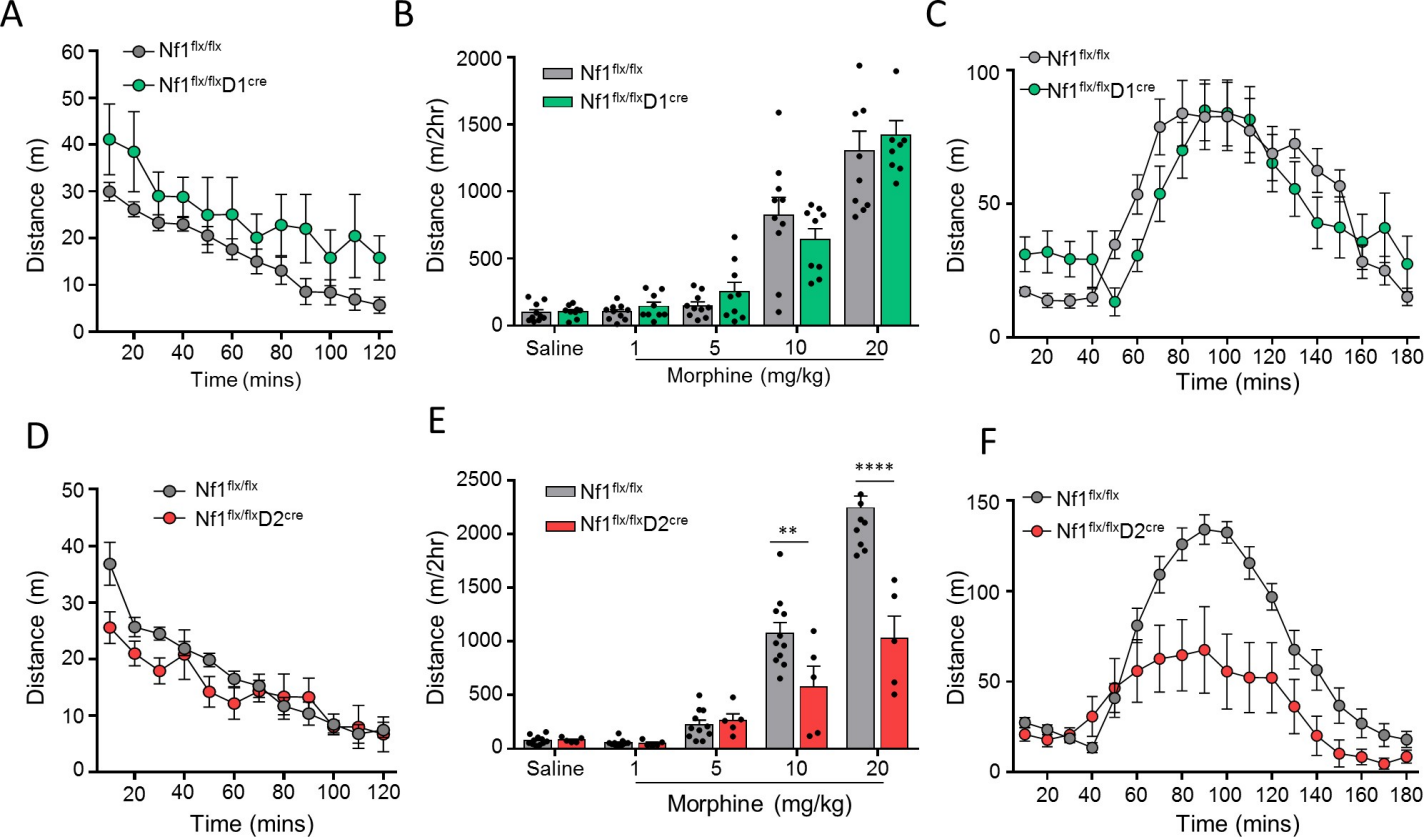
Elimination of NF1 in D2R-MSNs blunts morphine-induced locomotion. (A) Locomotor activity for naïve *Nf1*^*flx/flx*^*D1*^*Cre*^ mice. *n* = 5–10 mice/group. (B) Cumulative distance traveled in the open-field chamber for varying concentrations of morphine (1, 5, 10, and 20 mg/kg) and (C) time course for 10 mg/kg morphine for *Nf1*^*flx/flx*^*D1*^*Cre*^ mice. *n* = 9–10 mice/group. (D) Locomotor activity for naïve *Nf1*^*flx/flx*^*D2*^*Cre*^ mice. (E) Cumulative distance traveled in the open-field chamber for varying concentrations of morphine (1, 5, 10, and 20 mg/kg) and (F) time course for 10 mg/kg morphine for *Nf1*^*flx/flx*^*D2*^*Cre*^ mice. *n* = 5–11 mice/group. Data are represented as mean ± SEM. **P < 0.01, ****P < 0.0001, two-way RM ANOVA. Underlying data for this figure can be found in S1 Data. D2R, D2 dopamine receptor; MSN, medium spiny neuron; NF1, neurofibromin 1; RM-ANOVA, repeated measures analysis of variance.

Observations that NF1 acts only in D1R-MSNs to modulate opioid reward but controls morphine-induced changes in excitability in both MSN populations suggest that it may be involved in modulating other behaviors in D2R-MSNs sensitive to morphine exposure. To explore this possibility, we evaluated psychomotor effects of morphine. When tested in open field under drug-naïve conditions, *Nf1*^*flx/flx*^*D1*^*cre*^ mice exhibited no difference in basal locomotor activity compared with their *Nf1*^*flx/flx*^ littermates (Fig 3A). We found that administration of increasing doses of morphine enhanced motor activity in both *Nf1*^*flx/flx*^*D1*^*cre*^ and *Nf1*^*flx/flx*^ mice, with no significant differences between the genotypes in either extent or duration of the effects (Fig 3B and 3C). In contrast, while naïve *Nf1*^*flx/flx*^*D2*^*cre*^ mice had unchanged baseline locomotor activity (Fig 3D), they showed a markedly blunted effect to morphine-induced psychomotor activation (Fig 3E and 3F). Taken together, these findings suggest that NF1 may act in D2R-MSNs to control motor behaviors.

**Fig 4:**
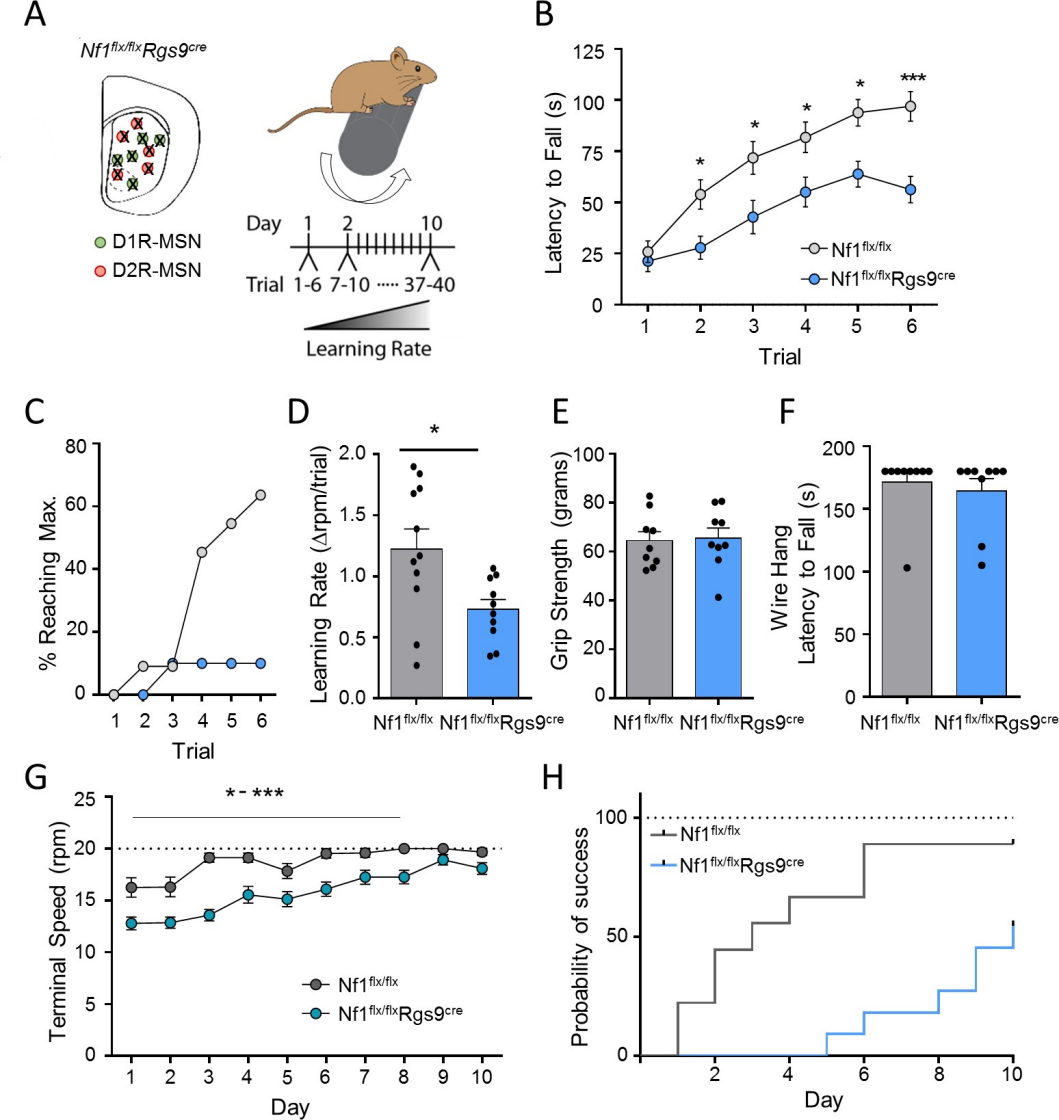
Key role of striatal NF1 in motor learning. (A) Scheme of NF1 deletion from MSNs and accelerating rotarod paradigm. (B) Performance of *Nf1*^*flx/flx*^ and *Nf1*^*flx/flx*^*Rgs9*^*cre*^ mice on the accelerating rotarod. *n* = 14 mice/group, two-way RM ANOVA. (C) Percentage of mice able to reach maximal speed. (D) Learning rate of *Nf1*^*flx/flx*^ and *Nf1*^*flx/flx*^*Rgs9*^*cre*^ mice on the accelerating rotarod. Student *t* test. (E) Grip strength and (F) wire hang for *Nf1*^*flx/flx*^ and *Nf1*^*flx/flx*^*Rgs9*^*cre*^ mice. *n* = 9 mice/group. (G) Time course of the terminal speed (trial 4) over 10 days of consecutive training on the accelerating rotarod for *Nf1*^*flx/flx*^ and *Nf1*^*flx/flx*^*Rgs9*^*cre*^ mice. *n* = 8–13 mice/group, two-way RM ANOVA. (H) Probability of reaching maximal speed over the 10 days of training on the accelerating rotarod. Data are represented as mean ± SEM. *P < 0.05, ***P < 0.001. Underlying data for this figure can be found in S1 Data. MSN, medium spiny neuron; NF1, neurofibromin 1; RM-ANOVA, repeated measures analysis of variance.

To further explore the contribution of NF1 in striatal-mediated movement control, we explored NF1’s role in motor learning. We started by evaluating the effect of pan-striatal NF1 deletion on performance in accelerating rotarod test deletion in *Nf1*^*flx/flx*^*Rgs9*^*cre*^ mice (Fig 4A). On the first trial, initial performance of *Nf1*^*flx/flx*^*Rgs9*^*cre*^ mice was indistinguishable from their *Nf1*^*flx/flx*^ control littermates, suggesting no effect on overall motor coordination (Fig 4B). However, over six consecutive sessions, performance of *Nf1*^*flx/flx*^*Rgs9*^*cre*^ mice improved significantly slower than that of control *Nf1*^*flx/flx*^ mice, as reflected by the time spent on the rotating rod (Fig 4B) and by the number of mice able to achieve maximum speed rotation (Fig 4C). This significant poor motor performance associated with NF1 loss is further reflected in the reduction of the learning rate (Fig 4D). Assays for innate reflectory motor behaviors such as grip (Fig 4E) and wire hang (Fig 4F) showed no genotype differences, further indicating the loss of NF1 specifically compromises their ability to learn a motor task rather than their overall physical ability.

To gain further insight into the trajectory of NF1 involvement in motor skill acquisition, we extended our studies to assess long-term motor learning across multiple daily sessions. As expected, control *Nf1*^*flx/flx*^ littermates progressively improved each day based on the latency time to fall (S5 Fig) and terminal speed (Fig 4G), with 88% of mice reaching a set criterion for at least one successful trial by day 6 (Fig 4H). In contrast, *Nf1*^*flx/flx*^*Rgs9*^*cre*^ mice exhibited severely protracted improvement in motor performance, as reflected in lower terminal speed (Fig 4G) and latency times during 10 training days (S5 Fig). While *Nf1*^*flx/flx*^*Rgs9*^*cre*^ mice eventually improved on the rotarod test, they required significantly more training than *Nf1*^*flx/flx*^ littermates to achieve a successful trial, with only 54% of *Nf1*^*flx/flx*^*Rgs9*^*cre*^ completing at least one successful trial during the entire training course (Fig 4H). Together, the data demonstrate that loss of striatal NF1 impedes motor learning by hindering the formation and consolidation of a simple repetitive motor routine.

**Fig 5:**
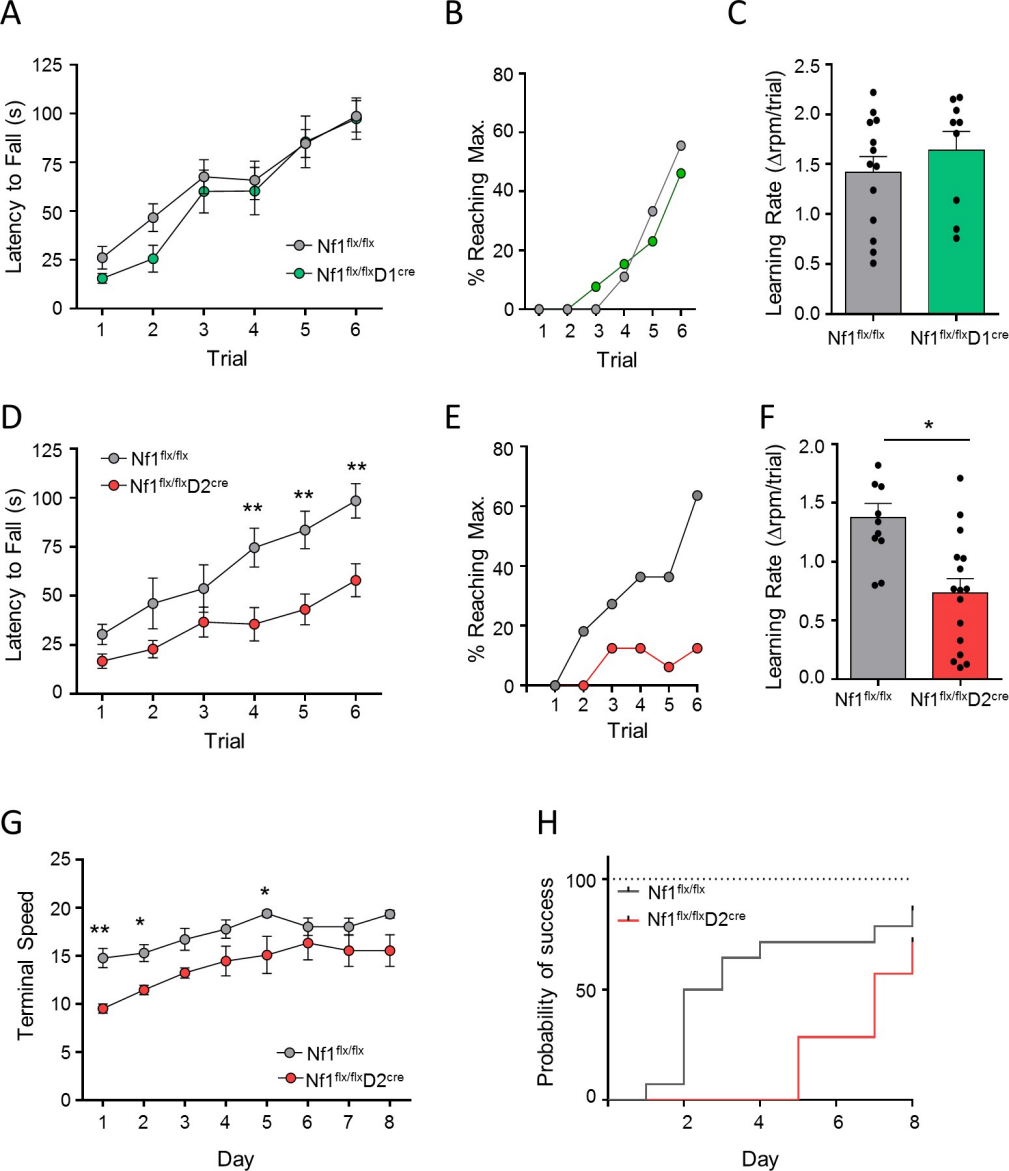
Action of NF1 in D2R-MSNs but not in D1R-MSNs is required for motor learning. (A) Performance of *Nf1*^*flx/flx*^ and *Nf1*^*flx/flx*^*D1*^*cre*^ mice on the accelerating rotarod. *n* = 9–13 mice/group. Two-way RM ANOVA. (B) Percentage of mice able to reach maximal speed and (C) learning rate on the accelerating rotarod for *Nf1*^*flx/flx*^ and *Nf1*^*flx/flx*^*D1*^*cre*^ mice. (D) Performance of *Nf1*^*flx/flx*^ and *Nf1*^*flx/flx*^*D2*^*cre*^ mice on the accelerating rotarod. *n* = 11–16 mice/group. Two-way RM ANOVA. (E) Percentage of mice able to reach maximal speed and (F) learning rate on the accelerating rotarod for *Nf1*^*flx/flx*^ and *Nf1*^*flx/flx*^*D2*^*cre*^ mice. (G) Time course of the terminal speed (trial 4) over 10 days of consecutive training on the accelerating rotarod for *Nf1*^*flx/flx*^ and *Nf1*^*flx/flx*^*D2*^*cre*^ mice. Two-way RM ANOVA. (H) Probability of reaching maximal speed over the 10 days of training on the accelerating rotarod. *P < 0.05, **P < 0.01. Data are represented as mean ± SEM. Underlying data for this figure can be found in S1 Data. D1R, D1 dopamine receptor; D2R, D2 dopamine receptor; MSN, medium spiny neuron; NF1, neurofibromin 1; RM-ANOVA, repeated measures analysis of variance.

Having established the role of NF1 in motor learning, we proceeded to elucidate the cell specificity of these effects using the same strategy by utilizing *D1*^*cre*^ and *D2*^*cre*^ drivers to selectively ablate NF1 in D1R-MSNs and D2R-MSNs, respectively. Consistent with the lack of effect on drug-induced locomotor activity, *Nf1*^*flx/flx*^*D1*^*cre*^ mice showed no difference in their performance on the rotarod when compared with *Nf1*^*flx/flx*^ littermates (Fig 5A and 5B), with both genotypes exhibiting similar learning rates (Fig 5C). In contrast, *Nf1*^*flx/flx*^*D2*^*cre*^ mice displayed decreased motor performance (Fig 5D), with a lower percentage of mice reaching a maximum rotational speed compared with *Nf1*^*flx/flx*^ mice (Fig 5E). *Nf1*^*flx/flx*^*D2*^*cre*^ mice also had a lower learning rate compared with *Nf1*^*flx/flx*^ mice (Fig 5F). Similar to pan-striatal NF1 deletion, no changes in grip strength or wire hang were observed in *Nf1*^*flx/flx*^*D2*^*cre*^ mice (S6A Fig and S6B Fig), again suggesting selective effects on motor learning and not due to physical ability. Assessing long-term motor performance, *Nf1*^*flx/flx*^*D2*^*cre*^ mice also required additional training days to match performance of wild-type littermates (Fig 5G, S6C Fig). Following 8 days of training, 72% of *Nf1*^*flx/flx*^*D2*^*cre*^ mice were able to achieve at least one successful trial, while this was reached in only day 4 for *Nf1*^*flx/flx*^ mice (Fig 5H). Overall, these findings show that NF1 acts in D2R-MSNs to support motor learning.

### Dopamine signaling to cAMP in MSN populations selectively relies on NF1

**Fig 6:**
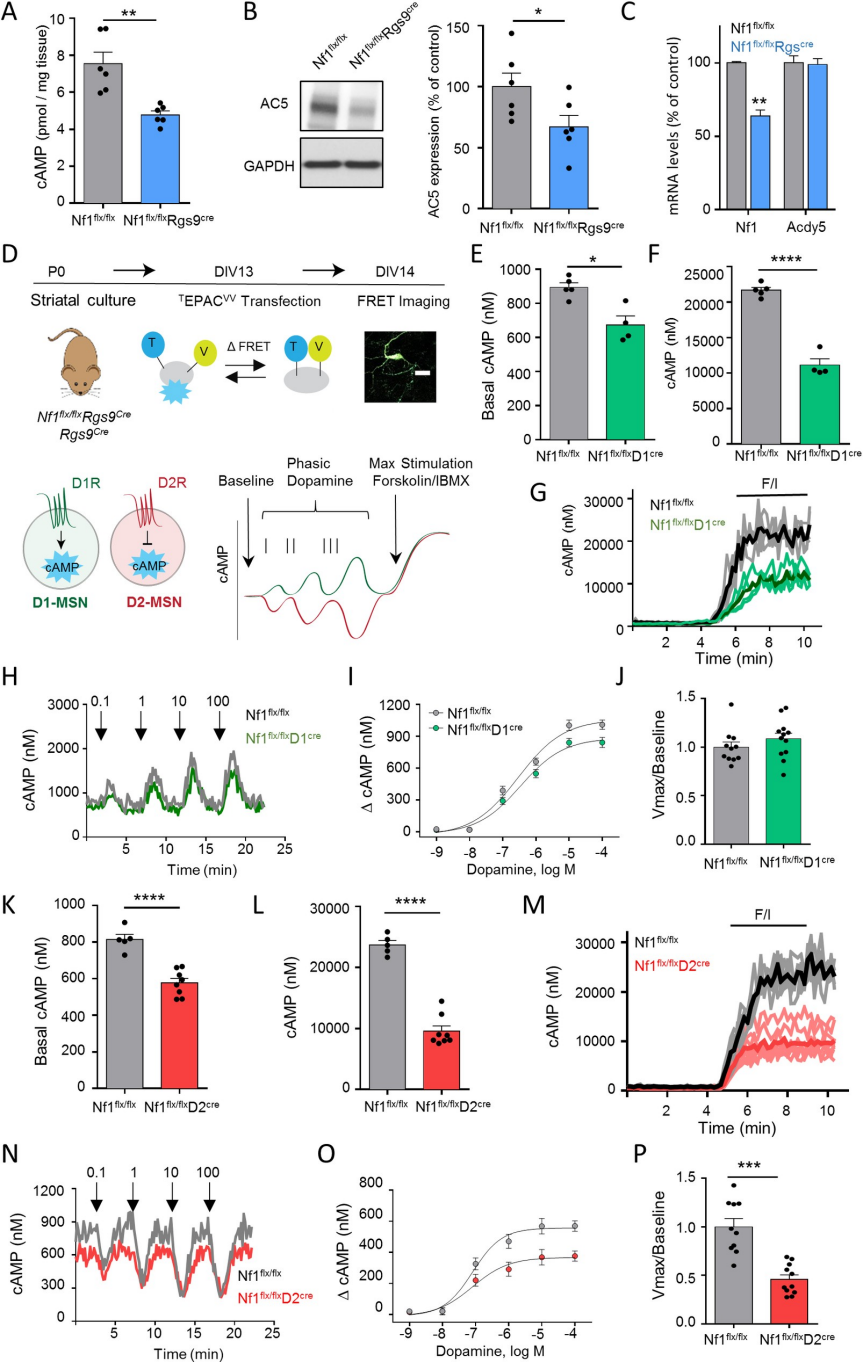
Cell-specific effects of NF1 on cAMP signaling in the striatum. (A) Quantification of total striatal cAMP in *Nf1*^*flx/*flx^ and *Nf1*^*flx/flx*^*Rgs9*^*Cre*^ mice. *n* = 6 mice/group, Student *t* test. (B) Representative image of western blot analysis for AC5 expression in the striatum of *Nf1*^*flx/flx*^ and *Nf1*^*flx/flx*^*Rgs9*^*Cre*^ mice. *n* = 6 mice/group. Student *t* test. (C) Comparison of mRNA for *Nf1* and *Adcy5* in striatum of *Nf1*^*flx/flx*^ and *Nf1*^*flx/flx*^*Rgs9*^*Cre*^ mice. *n* = 6 mice/group. Student *t* test. (D) Scheme and Image of ^T^EPAC^VV^ fluorescence in transfected primary MSNs. Mouse graphic was adapted from [44]. Scale bar represents 20 μm. Quantification of baseline striatal cAMP levels for (E) *Nf1*^*flx/flx*^*D1*^*Cre*^ and (K) *Nf1*^*flx/flx*^*D2*^*Cre*^ compared with *Nf1*^*flx/flx*^ mice. *n* ≥ 4 neurons/genotype. Change in cAMP following bath application of forskolin/IBMX to (F) D1R-MSNs and (L) D2R-MSNs. *n* ≥ 4 neurons/genotype. Quantification of maximum cAMP change following forskolin/IBMX treatment for (G) *Nf1*^*flx/flx*^*D1*^*Cre*^ and (M) *Nf1*^*flx/flx*^*D2*^*Cre*^ neurons. n ≥ 4 neurons per genotype. Representative traces of cAMP dynamics for (H) *Nf1*^*flx/flx*^*D1*^*Cre*^ and (N) *Nf1*^*flx/flx*^*D2*^*Cre*^ to a 1-second pulse of dopamine at the indicated micromolar concentration. Quantification of maximum change in cAMP response to phasic dopamine pulses for (I) *Nf1*^*flx/flx*^*D1*^*Cre*^ and (O) *Nf1*^*flx/flx*^*D2*^*Cre*^ neurons. Discrimination ratio (Vmax/Baseline) for cAMP response to dopamine in (J) *Nf1*^*flx/flx*^*D1*^*Cre*^ and (P) *Nf1*^*flx/flx*^*D2*^*Cre*^. *n* ≥ 4 neurons per genotype, Kolmogorov-Smirnov test. *P < 0.05, **P < 0.01, ***P < 0.001. Data are represented as mean ± SEM. Underlying data for this figure can be found in S1 Data. AC5, adenylyl cyclase type 5 (protein); *Adcy5*, adenylyl cyclase type 5 (gene); D1R, D1 dopamine receptor; D2R, D2 dopamine receptor; IBMX, 3-isobutyl-1-methylxanthine; MSN, medium spiny neuron; NF1, neurofibromin 1.

To determine the mechanism involved in motor learning mediated by NF1, we focused on the cAMP signaling pathway, which is differentially regulated by MSN populations. cAMP is known to be impacted by NF1 in other systems, yet this effect is poorly characterized and its underlying causes are unclear [38,47]. We found that ablation of NF1 in the striatum significantly diminished total striatal cAMP content (Fig 6A). We further observed concomitant reduction in expression of a major cAMP-producing enzyme in striatal neurons adenylyl cyclase type 5 (AC5) (Fig 6B), which is likely responsible for the lower baseline cAMP levels in *Nf1*^*flx/flx*^*Rgs9*^*cre*^ mice. Interestingly, *Adcy5* mRNA levels (transcript encoding AC5) were unchanged (Fig 6C), suggesting that down-regulation brought about by NF1 deletion is not a result of changes in transcription. The effect on AC5 expression was selective, and we detected no difference in the levels of regulatory G proteins: Gαolf or Gαo (S7A Fig).

We next explored the cellular mechanisms and neuronal specificity of this effect by studying the dynamics of cAMP signaling initiated by dopamine using a newly developed cAMP encoded reporter (CAMPER) imaging strategy for evaluating neurotransmitter signaling in striatal neurons [48]. In this approach, real-time changes in cAMP were recorded by Forster Resonance Transfer (FRET) by a genetically encoded sensor targeted selectively for D1R or D2R primary striatal neurons from *Nf1*^*flx/flx*^*D1*^*cre*^ and *Nf1*^*flx/flx*^*D2*^*cre*^ mice, respectively (Fig 6D). First, these measurements revealed reduction in basal cAMP levels in D1R-MSNs lacking NF1 as compared with control neurons (Fig 6E). No significant changes in AC5, Gαolf, or Gαo levels were found (S7B Fig). Application of forskolin together with IBMX, which increases cAMP by activating AC and by inhibiting PDE, respectively, increased cAMP levels; however, such increase was significantly blunted in D1R-MSNs lacking NF1 (Fig 6F and 6G), indicating reduction in the total AC content. Next, we probed responses of D1R-MSNs to phasic stimulation by dopamine. Application of dopamine puffs rapidly elevated cAMP levels, increasing response magnitude as the concentration of dopamine increased (Fig 6H). We observed no differences between *Nf1*^*flx/flx*^*D1*^*cre*^ and control *Nf1*^*flx/flx*^ neurons in net cAMP change across varying strengths of dopamine stimulation (Fig 6I). Furthermore, the signal discrimination ratio that reflects the degree of the maximal response relative to baseline remained unchanged upon NF1 loss in D1R-MSNs (Fig 6J).

We observed similar reduction in baseline cAMP levels, total activatable AC content, and AC5 levels with no change in Gαolf or Gαo levels in D2R-MSNs upon elimination of NF1 (Fig 6K–6M, S7C Fig). In contrast to D1R-MSNs, responses of D2R-MSNs to phasic dopamine were markedly affected (Fig 6N). This was reflected in significantly diminished response amplitudes in *Nf1*^*flx/flx*^*D2*^*cre*^ as compared with *Nf1*^*flx/flx*^ controls across varying dopamine concentrations (Fig 6O), resulting in compression of the signal discrimination ratio (Fig 6P). Altogether, these experiments demonstrate that while loss of NF1 reduced baseline cAMP levels in all striatal neurons, the deficits in processing dopamine-mediated changes in cAMP selectively affected the D2R-MSNs.

### Restoration of cAMP in D2R-MSNs rescues motor learning deficits associated with NF1 loss

**Fig 7:**
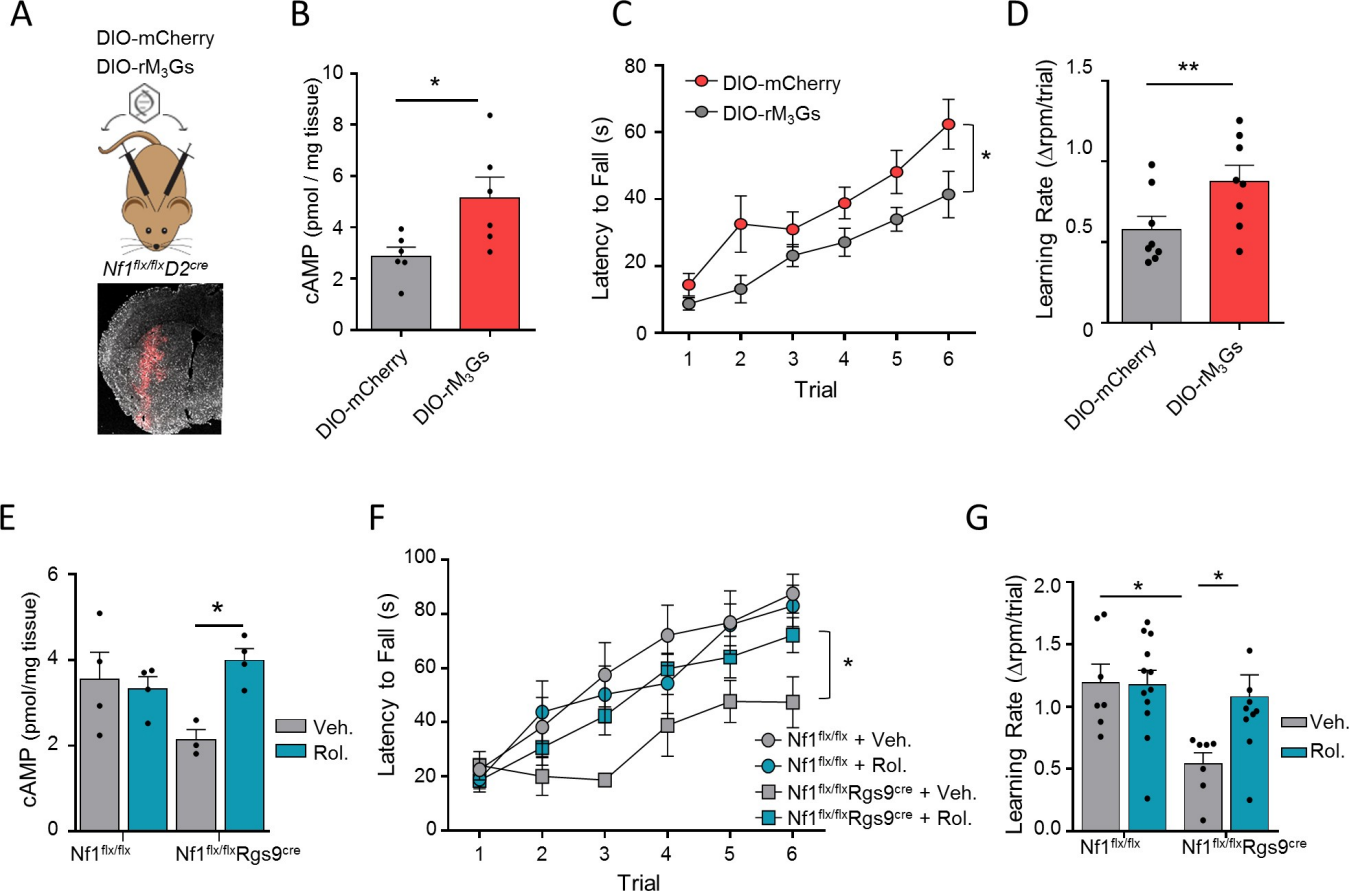
Chemogenetic and pharmacological rescue of motor deficits associated with NF1 deficiency. (A) Scheme and Image of *Nf1*^*flx/flx*^*D2*^*cre*^ mice bilaterally injected in the striatum with AAV-DIO-rM_3_Gs. Mouse graphic was adapted from [44]. (B) Quantification of striatal cAMP levels of *Nf1*^*flx/flx*^*D2*^*cre*^ bilaterally injected with AAV-DIO-rM_3_Gs or DIO-mcherry upon CNO administration. *n* = 6 mice/group. (C) Increase in performance and (D) learning rate on the accelerating rotarod of DIO-rM_3_Gs mice compared with DIO-mcherry mice following CNO injection in *Nf1*^*flx/flx*^*D2*^*cre*^ mice. *n* = 6–7 mice/group. (E) Quantification of striatal cAMP levels in *Nf1*^*flx/flx*^ and *Nf1*^*flx/flx*^*Rgs9*^*cre*^ treated with rolipram. (F) Performance and (G) learning rate on the rotarod task for *Nf1*^*flx/flx*^ and *Nf1*^*flx/flx*^*Rgs9*^*cre*^ mice treated with rolipram. *n* = 7–10 mice/group. Two-way RM ANOVA. *P < 0.05, **P < 0.01. Data are represented as mean ± SEM. Underlying data for this figure can be found in S1 Data. DIO, double-floxed inverted open reading frame; NF1, neurofibromin 1; RM-ANOVA, repeated measures analysis of variance; Rol, rolipram; Veh, vehicle.

Our observations suggest that decreased neurotransmitter signaling to cAMP in D2R-MSNs may be responsible for motor learning impairments upon NF1 loss. To test this hypothesis, we examined whether elevation of cAMP levels in D2R-MSNs can rescue motor learning deficits by utilizing rM_3_Ds designer receptor exclusively activated by designer drug (Designer Receptors Exclusively Activated by Designer Drugs [DREADD]), which couples to Gs/olf to stimulate cAMP production when activated by its ligand [49]. We bilaterally injected a Cre-dependent AAV8-hSyn-DIO-hM_3_Ds-mCherry (DIO-M_3_Ds) or control AAV8-hSyn-DIO-mCherry (DIO-Cherry) into the striatum of *Nf1*^*flx/flx*^*D2*^*cre*^ mice (Fig 7A) and then tested them in the rotarod task. Following surgeries, mice were treated with clozapine-N-oxide (CNO) to activate the DREADD, which caused a significant increase in cAMP levels (Fig 7B). Remarkably, we found that administration of CNO to *Nf1*^*flx/flx*^*D2*^*cre*^ mice injected with AAV8-hSyn-DIO-hM3Dq-mCherry, but not with control AAV8-hSyn-DIO-mCherry virus, enhanced their motor performance and improved learning rate (Fig 7C and 7D). Control experiments showed that injection of DIO-M_3_Ds or control DIO-mCherry in *Nf1*^*flx/flx*^*D2*^*cre*^ mice without CNO treatment did not have an effect on rotarod performance of mice (S8A Fig and S8B Fig) or on locomotor activity in open field (S8C Fig and S8D Fig). We also tested whether CNO administration would affect performance on the rotarod assay. There was no difference in performance or learning rate between CNO and vehicle treated mice (S8E Fig and S8F Fig). These findings indicate that restoration of cAMP responsiveness in D2R-MSNs of NF1-deficient mice is sufficient to rescue their motor learning deficits.

To explore the translational relevance of these observations, we next increased cAMP levels pharmacologically by administering a PDE inhibitor, rolipram, a chosen target for its clinical efficacy [50]. The chosen dose of rolipram increased cAMP levels in the striatum of *Nf1*^*flx/flx*^*Rgs9*^*cre*^ mice (Fig 7E). While rolipram treatment had no effect on motor performance in control *Nf1*^*flx/flx*^ mice, it significantly increased the overall performance of *Nf1*^*flx/flx*^*Rgs9*^*cre*^ mice by completely rescuing their motor learning deficits (Fig 7F and 7G). On day 3 of training, 50% of *Nf1*^*flx/flx*^*D2*^*cre*^ mice treated with rolipram were able to achieve at least one successful trial, similar to control *Nf1*^*flx/flx*^ mice (S9A Fig). Similarly, rolipram-treated *Nf1*^*flx/flx*^*D2*^*cre*^ mice showed increased motor performance compared with *Nf1*^*flx/flx*^ mice (S9B Fig and S9C Fig). Thus, strategies aimed at increasing cAMP levels may be useful in ameliorating motor deficits associated with NF1 loss.

## Discussion

Here, we report that contributions of D1R-MSNs and D2R-MSNs to key striatal-mediated behaviors can be dissociated at the molecular level by NF1. Using a mouse model featuring the loss of the multifunctional signaling protein NF1, we show that populations of striatal neurons differentially contribute towards morphine-induced reward and motor learning behaviors. We found that NF1 acts in D2R-MSNs to enable motor learning and morphine-induced locomotion, whereas its action in D1R-MSNs sets the sensitivity for reward valuation. Remarkably, we observed that NF1 selectively impacts the processing of neurotransmitter inputs via distinct signaling pathways. Ablation of NF1 decreased cAMP in both MSN populations but only D2R-MSNs showed deficits in dopamine-mediated cAMP changes. Furthermore, by rescuing cAMP in D2R-MSN, motor learning deficits improved in our NF1-deficient mice model. Taken together with the selective role of NF1 in controlling opioid signaling to Ras only in D1R-MSNs [31], these observations suggest a model that NF1 routes signals initiated by neurotransmitter GPCRs to distinct intracellular pathways (cAMP versus Ras). The relative impact of NF1 on cAMP or Ras signaling is then dependent on the identity of the neuronal cell type. Intriguingly, this suggests that the mechanism for multiplexing signals originates at GPCRs by increasing their signaling capacity and allowing the same neuromodulatory input to control various striatal-mediated behaviors.

The striatum has a fundamental role in learning goal-oriented behaviors that is driven by repeating a motor task, in which each neuronal population contributes to the learning process. Recent studies have shown that direct and indirect pathways work in tandem to regulate movement [46,51] and are differentially modulated during motor performance [52]. In extended motor training in which consolidation of the motor action is occurring, D2R-MSNs have been shown to be predominantly engaged and become less dependent on the activation of D1R-MSNs. By investigating the performance after initial and prolonged training of mice in the accelerated rotarod task, we were able to assess the acquisition and consolidation of motor learning and identified NF1 in D2R-MSNs as a molecular component responsible for consolidating motor action. With prolonged training, these mice were able to learn this motor task, indicating they are able to establish a basic action sequence but require significantly more training sessions to reach set criteria. This response was also recapitulated during the food training in operant chambers, where mice learn to lever press. Additional training has also been shown to improve learning performance of NF1-deficient mutants in the spatial learning paradigms [34]. The limitations associated with the choice of the Cre-driver lines used in this study should be noted. In particular, both *D2*^*cre*^ and *Rgs9*^*cre*^ target striatal CINs, while *D2*^*cre*^ additionally targets dopaminergic midbrain neurons. As NF1 is also expressed in these neuronal populations, we cannot completely rule out any potential contributing effects of NF1 in these neurons on motor learning. These concerns may be partially mitigated by the lack of observable changes in excitability of CINs in *Nf1*^*flx/flx*^*D2*^*cre*^, in contrast to clearly affected MSNs in our NF1-deficient models. Future studies will be needed to further investigate the specific role of NF1 in these interneurons, especially at the behavioral level. The loss of NF1 in D1R-MSNs had a similar learning rate to wild-type animals in the rotarod task, but established lever pressing in operant food-training earlier. In fact, during the initial sessions, *Nf1*^*flx/flx*^*D1*^*cre*^ mice significantly pressed more levers and most mice reached the set criteria, indicating that these mice established this motor skill during the early acquisition phase. We suggest that an effect in the rotarod could not be identified, given that the mice likely hit a ceiling effect, a known caveat of this basic motor task given its simplicity. Although we may not be able to rule out the potential rewarding effect of lever pressing, it is in contrast to their blunted behavioral response in the reward paradigms. While we have distinguished cell-specific roles for NF1 in MSN, there may also be regional specificity for NF1 effects (i.e., dorsal versus ventral striatum). Overall, NF1 appears to be a molecular determinant for the consolidation of motor learning actions within the indirect pathway.

In this study, we show that control of motor learning by NF1 is dependent on cAMP signaling. Investigating this influence, we further obtained significant insight into the connection of NF1 to the cAMP signaling axis. The impact of NF1 on cAMP production has been well noted, but the molecular mechanisms of this regulation have been ill-defined [39–41]. Notably, GPCR-mediated regulation of cAMP by NF1 has been demonstrated to be independent of its RasGAP activity [53], and our data further support this idea. We found that NF1 plays a role in controlling the expression level of cAMP-producing enzyme, AC5, likely by affecting its posttranslational stability, as it did not impact the transcription. As a result, AC5 deficiency sets lower levels of basal cAMP tone, compressing the range of the inhibitory response in D2R-MSNs and compromising processing of phasic dopamine signals. Consistent with this model, raising baseline cAMP levels in D2R-MSNs ameliorated the motor learning deficits brought about by NF1 loss. Interestingly, recent studies suggest that pharmacological activation of the cAMP pathway may enhance cognition in general murine models [54,55] as well as visually guided habituation learning in a NF1-defficient zebrafish model [37]. In light of this additional evidence, it seems plausible that learning deficits in human patients with NF1 may at least in part be due to perturbations of cAMP regulation by GPCRs. Given that preclinical trials of Ras–mitogen-activated protein kinase (MAPK) inhibitors, lovastatin and simvastatin, failed to improve learning and attention deficits in children with NF1 [56,57], cAMP signaling enhancement strategies may be a promising avenue for ameliorating the cognitive dysfunction in NF1.

Concerted efforts are being made to decipher NF1 signaling mechanisms in order to better understand how its disruption causes disease. Along this line, several genetic models with mutations in the orthologs of the human *NF1* gene have been created and shown to recapitulate many aspects of the disease, including tumor formation and cognitive and behavioral impairments [58,59]. For instance, *Nf1* haploinsufficient (*Nf1*^+/–^) mouse model has shown impaired spatial learning and memory in the Morris water maze and social recognition deficits [36,60], as well as impairments in early social communicative behaviors [61]. Interestingly, some of these behavioral deficits have been attributed to altered striatal dopamine levels [62]. A conditional NF1 deletion in neuroglial progenitor cells (*Nf1*^+/–GFAP^ conditional knockout mice) was originally developed to model optic pathway gliomas [63], but these mice also display attention and memory deficits that result from reduced striatal dopamine levels [42,64,65]. Patient data indicate that reduced neurofibromin can lead to dysregulated dopamine levels, which may contribute to the observed attentional and learning impairments in children with NF1 [62]. Our data provide another layer by demonstrating that NF1’s ability to process dopamine signaling is cell specific. These observations are interesting to consider in light of promise that dopamine reuptake inhibitors are showing in early clinical trials, ameliorating behavioral and cognitive deficits in children with NF1 [66,67]. Overall, identifying signaling derivations caused by NF1 mutations may prove to be instrumental in understanding clinical manifestations of the NF1 disorder.

Another major observation of this work is that both D1R-MSNs and D2R-MSNs display decreased intrinsic excitability in response to repeated morphine administration. Loss of NF1 prevented this neuroadaptation while having no effect on baseline excitability in naïve mice, suggesting a selective role of NF1 in adaptive response. The implication of NF1 in regulating excitability in striatal neurons is consistent with its effects on cAMP and a critical role of this second messenger in modulating excitability in the striatum [68,69]. Previous studies have reported no effect of NF1 on the excitability of hippocampal pyramidal neurons but found increased excitability of parvalbumin-positive interneurons in hippocampus as well as nociceptive sensory neurons [70,71], further emphasizing the point of NF1’s cell-specific effects. While morphine-induced changes in excitability of striatal neurons that we report were similar in both MSN populations, behavioral outcomes differed, suggesting the role of NF1 in integrating distinct signaling inputs. Because NF1 is involved in MOR-mediated signaling to Ras exclusively in D1R-MSNs [31] and, as we report here, D1R-MSNs’ pathway is sufficient to regulate opiate induced reward-related behaviors, these observations suggest that NF1 regulates morphine reward behavior via Ras signaling pathway in D1R-MSNs. In addition, our results suggest that NF1 integrates signaling from the D2R to the cAMP pathway, controlling the motor learning process selectively in the D2R-MSN population. Thus, NF1 serves a dual role in both multiplexing GPCR signaling, parsing out their effects on downstream effectors in a cell collective manner to regulate discrete behavioral outcomes.

## Methods

### Ethics statement

The animal studies were carried out in accordance with the National Institutes of Health guidelines and were granted formal approval by the Institutional Animal Care and Use Committee of the Scripps Research Institute (approved protocol #16–032).

### Animals

Generation of *Nf1*^*flx/flx*^ and *Rgs9*^*cre*^ mice was described previously [31,72]. *D1*^*cre*^ (Drd1-Cre; EY262; stock #017264-UCD), *D2*^*cre*^ (Drd2-Cre; ER43; Stock #017268-UCD), and Drd2-GFP (S118Gsat) were obtained from MMRRC. Conditional knockout mice were generated by crossing *Nf1*^*flx*^ mice with *Rgs9*^*cre*^, *D1*^*cre*^, or *D2*^*cre*^ for two generations to generate homozygous *Nf1*^*flxP/flx*^*Rgs9*^*cre*^, *Nf1*^*flx/flx*^*D1*^*cre*^, and *Nf1*^*flx/flx*^*D2*^*cre*^ knockout mice and their wild-type littermate control *Nf1*^*flx/flx*^ mice. All mice are on the C57/Bl6 background and relied exclusively on littermates for all the comparisons. Genotyping was used to monitor the status of all alleles in all mice used in experiments. For genotyping, DNA was extracted from ear punches and PCR analysis was conducted using standard techniques. Genotyping primer sequences are as follows: Cre lines: Fwd-5′ GCG GTC TGG CAG TAA AAA CTA TC, Rev-5′ GTG AAA CAG CAT TGC TGT CAC TT; *Nf1*^*flx/*flx^: WT-5′ ACC TCT CTA GCC TCA GGA ATG A, Mutant-5′ TGA TTC CCA CTT TGT GGT TCT AAG, common-5′ CTT CAG ACT GAT TGT TGT ACC TGA. Male mice were used for all behavioral tests while both male and female mice were used for biochemistry analysis, and were between 2 and 4 months of age. Mice were housed in groups on a 12-hour light–dark cycle with food and water available ad libitum.

### Rotarod

Rotarod performance was tested using a five-station rotarod treadmill (IITC Life Sciences, Woodland Hills, CA) with an acceleration from 8 to 20 rpm. Rotarod testing consisted of six trials per day with 5 minutes between intertrial intervals, while daily testing consisted of four trials per day up to 10 consecutive days. Mice were returned to the home cage in between trials. Each trial ended when a mouse fell off the rod, completed one full revolution on the rod, or reached 120 seconds and the time was scored as the latency to fall. For rolipram and CNO experiments, mice were handled and injected with vehicle (IP) for 3 days in order to minimize stress. On test day, mice were injected and placed back into their home cage, and the rotarod test was performed 30 minutes following injection, consisting of six trials with 5 minutes between intertrial intervals.

### Grip strength tests

Grip strength was measured as the peak force using a grip strength meter (Ugo Basile Italy). The mice were handled by their tails and placed over the grid until both forearms grasped the grid. The tail was then pulled horizontally until the mouse released hold entirely. Three separate readings were recorded for each mouse, with a corresponding 20 seconds between each trial.

### Locomotion

Locomotor testing was conducted as previously described [73]. Briefly, locomotor activity was performed in 40 × 40 × 35-cm chambers (Stoelting Co, Wood Dale, IL), and distance traveled was recorded using Anymaze video-tracking software. Under naïve conditions, mice were placed in the center of the chambers, and distance traveled was measured for 2 hours and analyzed in 10-minute bins. For pharmacological experiments, mice were handled and injected with vehicle for 3 days in order to minimize stress. For morphine experiments, mice were allowed to acclimatize to the chambers for 30 minutes before being administered morphine (s.c.) at a concentration of 1 mg/kg, 5 mg/kg, 10 mg/kg, or 20 mg/kg or saline and were recorded for 3 hours.

### CPP

CPP was conducted using a two-chamber box with a tunnel adjoining the chambers, in which each chamber was distinguished by different color and floor texture (Stoelting Co, Wood Dale, IL), and was performed as previously described [73]. The CPP procedure consisted of four phases: habituation, preconditioning test, conditioning, and post-conditioning test. Time spent in each chamber was measured during each phase of the CPP using video tracking followed by the analysis by Anymaze Software (Wood Dale, IL). Animals were first habituated to the apparatus by allowing free access to all compartments for 20 minutes. The following day, all mice were exposed to a 30-minute preconditioning phase, during which each animal was given free access to the CPP apparatus. Overall, mice did not show a preference for either side of the chamber during the preconditioning and consequently the drug side was randomly assigned. During the 6 days of conditioning, animals were injected once a day with either vehicle or morphine (1 or 10 mg/kg, s.c.) and immediately confined to one of the assigned compartments for 30 minutes. The order of the drug administration was counterbalanced such that half the animals received morphine on the first day of conditioning and the other half on the second day of conditioning. The day after the last conditioning, mice were allowed free access to all compartments for 30 minutes (post-conditioning). Place preference score was calculated for each mouse as the difference between post-conditioning and preconditioning time spent in the drug-paired compartment.

### Self-administration

Behavioral training occurred in an operant conditioning chamber (14 × 12.7 × 16 cm; Med Associates, St. Albans, VT) housed inside standard sound-attenuating cubicles (56 × 38 × 40.6 cm). The self-administration paradigm consisted of food self-administration training to ensure that all mice learned to lever press before being permitted to acquire intravenous morphine self-administration and has been previously described [73–75]. For food self-administration, mice were first acclimated to food pellets, which were subsequently used to reinforce operant responding. Mice were fasted overnight, maintaining 85% of pre-fasted body weight and were trained to self-administer food pellets under a fixed-ratio 1 (FR1) schedule with a time-out period of 20 seconds (FR1 TO20) in 1-hour daily sessions. Mice were gradually trained to FR5 TO20, and then they underwent surgery for jugular catheter implantation. Following post-surgery recovery, mice learned to self-administer morphine (0.3 mg/kg/infusion), defined by a minimum of 15 infusions/session for 3 consecutive days, with a minimum response ratio of 5:1 between active and inactive levers. They were then placed on a dose-response schedule of 0.6, 1.0, and 0.1 mg/kg, returning to a baseline dose of 0.3 mg/kg to ensure proper response. Data were expressed as the number of lever presses in a session or the mean number of infusions of the last three stable sessions at each dose of morphine.

### Stereotaxic injections

AAV8-hSyn-DIO-rM3D(Gs)-mCherry and AAV8-hSyn-DIO-mCherry were obtained from Duke Vector Viral Core, and AAV9-CAGLex.eGFP.WPRE.bGII was purchased from the Allen Institute. Mice were anesthetized with ketamine (90 mg/kg IP) and xylazine (10 mg/kg IP) and placed in a stereotaxic device, where AAVs were bilaterally microinjected using a Hamilton microsyringe. AAV9-CAGLex.eGFP.WPRE.bGII (0.3 mL per side) was delivered into the NAc using the stereotaxic coordinates from bregma: anteroposterior, +1.1 mm; mediolateral, 1.0 mm; dorsoventral, 3.8 mm. AAV8-hSyn-DIO-rM3D(Gs)-mCherry and AAV8-hSyn-DIO-mCherry (0.6 mL per side) were delivered into the striatum using the stereotaxic coordinates from bregma: anteroposterior, +0.7 mm; mediolateral, 1.95 mm; dorsoventral, 2.8 mm. The injection rate was 0.3 mL over 5 minutes, and the injectors were kept in place for an additional 5 minutes to ensure adequate diffusion from the injector tip. Mice were allowed to recover for 2–3 weeks before any behavioral testing was conducted.

### Drugs

Rolipram was purchased from Tocris and CNO was purchased from Cayman Chemical Company. Drug stocks were prepared with DMSO, in which the final DMSO for rolipram was 0.3% and for CNO was 1.0%. Vehicle injections were the respective DMSO concentrations (0.3% or 1.0%). For behavioral experiments, mice were handled and injected with vehicle for 3 days in order to minimize stress.

### Biochemical cAMP quantification

Striatal tissue punches were flash frozen in liquid nitrogen followed by homogenization in ice-cold buffer consisting of 20 mM HEPES pH 8.0, 1 mM EDTA, 150 mM NaCl, 2 mM MgCl_2_, 1 mM dithiothreitol, and 1× complete protease inhibitor (Roche, Indianapolis, IN) followed by centrifugation at 610*g* for 10 minutes. For pharmacological experiments, mice were injected with 1 mg/kg CNO (IP) or rolipam (0.3 mg/kg IP), and striatal tissue punches were taken 30 minutes postinjection. The supernatant was diluted 1:50 in 0.1 M HCl and the total amount of cAMP was quantified by immunoassay ELISA according to the manufacturer’s acetylated protocol (Direct cAMP ELISA Kit, ENZO Life Sciences, Farmingdale, NY). Plate absorbance was recorded at 405 nm on a PHERAstar FSX instrument (BMG Labtech, Cary, NC) and data were normalized to tissue mass.

### Primary culture

Striatal neurons were cultured as previously described with slight modification [31]. Brains from postnatal day 0 pups were rapidly removed and striata were quickly dissected in ice-cold HBSS supplemented with 20% FBS, 4.2 mM NaHCO_3_, and 1 mM HEPES. Tissue was washed three times in HBSS solution without FBS and digested in a 37°C water bath for 20 minutes in a solution consisting of 137 mM NaCl, 5 mM KCl, 7 mM Na2HPO_4_, 25 mM HEPES, and 0.3 mg/mL Papain at pH 7.2. The tissue was then washed three times in HBSS/FBS, three times in HBSS without FBS, and three times in growth media that consisted of Neurobasal-A supplemented with 2 mM GlutaMAX, 2% B27-supplement, and 1% PenStrep. The tissue was triturated by pipette in growth media supplemented with 0.05 U/μL DNAse I, filtered by a 40-μm cell strainer, and plated on poly-D-lysine–coated glass coverslips. Cultures were grown at 37°C in a 5% CO_2_ humidified incubator, whereupon half of the media was removed and replenished every 3 days with media that did not contain PenStrep. Neurons were transfected with ^T^EPAC^VV^ [76] on DIV 13 using Lipofectamine 2000.

### FRET imaging

All images were obtained on a Leica TCS SP8 MP confocal microscope through a 25× water immersion objective lens. A 442-nm diode laser was used for excitation of mTurquoise that was paired with simultaneous 465–505-nm (mTurquoise) and 525–605-nm (Venus) band-pass emission filtration. Images were captured at 10-second intervals that consisted of multiple Z-stacks. Fluorescence intensity from neuronal cell bodies were quantified using ImageJ to calculate the FRET ratio. Drugs were either bath applied or administered in phasic 1-second pulses through an SF-77B perfusion apparatus (Warner Instruments, Hamden, CT) as indicated in the text. dMSNs and iMSNs were identified by directionality of the response to dopamine. Conversion of FRET values to nanomolar cAMP values was performed as described utilizing the calibration curve of the ^T^EPAC^VV^ sensor in response to defined cAMP standards. The standard curve was generated by permeabilizing live neurons with high-purity digitonin (10 μg/mL) to deplete intracellular cAMP content. Standardized cAMP solutions were then bath applied while recording the ^T^EPAC^VV^ biosensor FRET response, which remained stable for at least four minutes. A nine-point concentration response curve was generated from the cAMP-induced FRET response with an EC50 of 3.0 ± 0.5 μM, similar to other reports utilizing Epac1-based biosensors [77,78]. Prism GraphPad 6 was used to interpolate experimental values to nanomolar values from this standard curve. Δ cAMP was calculated as the difference between peak cAMP change and the baseline. Signal to background was calculated as the cAMP amplitude divided by the baseline cAMP value. For experiments in which the cAMP signal decreased upon stimulation, experimental values were inverted to generate signal to noise calculation.

### Quantitative real-time PCR

Total RNA from the striatum of *Nf1*^*flx/flx*^ and *Nf1*^*flx/flx*^*Rgs9*^*Cre*^ mice was extracted using TRIZOL reagent (Invitrogen) according to the manufacturer's instructions. RNA in the aqueous phase was further purified with RNeasy spin column (Qiagen), and its concentration was measured with a NanoDrop spectrophotometer (Thermo Fisher Scientific). Reverse transcription was carried out using qScript cDNA Supermix (Quantabio) for qRT-PCR from 610 ng of total RNA. The analysis of RNA expression of the target genes was performed on a 7900HT Fast Real-Time PCR System (Applied Biosystems) with Taqman probes under the following conditions: 95°C for 10 minutes, followed by 40 cycles of 95°C for 15 seconds, 60°C for 1 minute. Four biological replicates and three technical replicates for each sample were used. A total of 16 ng of each sample was used in each real-time PCR (TaqMan Gene Expression Assay ID probe: *Adcy5*, Mm00674122_m1; Nf1, Mm00812424_m1; Applied Biosystems). The expression ratio of the target genes was calculated using the *Gapdh* (ID: Mm99999915_g1) as reference using the 2^−ΔΔCT^ method [79].

### Western blotting

Tissue punches of the striatum were homogenized in ice-cold buffer (137 mM NaCl, 20 mM Tris [pH 8.0], 1% NP-40, 10% glycerol, and 0.1% sodium dodecyl sulfate), with the addition of protease and phosphatase inhibitors (Roche, Rockford, IL), and then sonicated. Protein concentration of tissue lysates was determined by Pierce 660 nm Protein Assay Reagent (Thermo Fisher, Waltham, MA); samples were diluted to the same concentration and then denatured in 5× SDS sample buffer (pH 6.8) for SDS-PAGE analysis. Following transfer to PVDF membranes, the membranes were blocked in 5% nonfat dry milk dissolved in Tris-buffered saline + 0.05% Tween 20 (TBST) for 1 hour at room temperature (22–26°C). To detect the proteins of interest, membranes were incubated with the following primary antibodies: anti-AC5 (1:3,000) [80], NF1 (Bethyl Laboratory, 1:1,000) [31], Gαo (Cell signaling, cs-3975, 1:1,000), Gαolf [81], sos1 (Santa Cruz Biotechnology, sc-10803; 1:200), RasGRF2 (Santa Cruz Biotechnology, sc-7591; 1:100), p120GAP (BD Biosciences, 610040; 1:1,000), SynGAP (Millipore, 06–900; 1:4,000), RasGRP1 (Santa Cruz Biotechnology sc-365358, 1:100), and anti-GAPDH (Millipore AB2302, 1:25,000). Following incubation with primary antibodies, the membranes were washed three times with TBST and then incubated with species-specific HRP-conjugated secondary antibody solution (Jackson ImmunoResearch Laboratories, Mouse Anti-Rabbit 211-032-171, 1:75,000; Goat Anti-Mouse 115-035-174, 1:75,000). To visualize the protein of interest, membrane was exposed to SuperSignal West Femto or West Pico substrate (Thermo Fisher, Waltham, MA) and signal was captured on film (Kodak X-Omat LS). Band density was measured using ImageJ software.

### Electrophysiology

Mice were euthanized under isoflurane anesthesia, and brains were rapidly removed and placed in ice-cold cutting solution composed of (mM): 125 Choline Cl, 2.5 KCl, 25 NaHCO_3_, 1.25 NaH_2_PO_4_, 5 MgSO_4_, 0.5 CaCl_2,_ and 10 D-Glucose, equilibrated with 95% O_2_ and 5% CO_2_. The tissue was cut in 300-μm-thick coronal sections with a Vibrating microtome (Leica VT1200S, Germany). The slices were maintained for 1–6 hours in artificial cerebrospinal fluid (aCSF) composed of (mM) the following: 119 NaCl, 2.5 KCl, 26.2 NaHCO_3_, 1 NaH_2_PO_4_, 1.3 MgSO_4_, 2.5 CaCl_2_, and 11 D-Glucose, equilibrated with 95% O_2_ and 5% CO_2_. After recovery for at least 1 hour, slices were transferred to a submerged-type recording chamber of approximate volume 1 mL. Here, the slices were constantly superfused (1–2 mL/minute) with warmed (30–31°C), gassed aCSF containing 100 μM picrotoxin. All measurements were performed by an experimenter blind to genotype or condition.

Core MSNs were visually identified in the slices using Scientifica SliceScope system. Membrane potentials and whole-cell currents were measured with hardware (Axopatch-700B amplifier, Digidata 1440A) and software (pCLAMP v. 10.3) from Molecular Devices (Sunnyvale, CA). All currents were low-pass filtered at 2 kHz, sampled at 10 kHz, and stored on computer hard disk for subsequent analysis. Glass microelectrodes with an open-tip resistance of 3–5 MΩ were used. The following internal solutions were used (mM) for current clamp experiments: 120 K-Gluconate, 20 KCl, 2 MgCl_2_, 10 K-HEPES, 0.2 EGTA, 0.3 Na_3_-GTP, and 4 Na_2_-ATP (pH 7.3). To determine intrinsic cellular properties such as RMP, input resistance, and spike numbers, 800-ms, 25-pA, multiple-step hyperpolarizing and depolarizing current injections were delivered every 10 seconds. Cells with series resistance >35 MΩ or with >20% change in series resistance were excluded from analysis. To record AMPAR/NMDAR currents, coronal slices (300 μm) containing striatum (AP + 0.9–1.7) were cut in ice-cold aCSF (in mM: 124 NaCl, 2.8 KCl, 1.25 NaH_2_PO_4_, 2 CaCl_2_, 1.25 MgSO_4_, 26 NaHCO_3_, 10 glucose, pH 7.5, bubbled with 95% O_2_/5% CO_2_) using a vibrating tissue slicer (VT1200, Leica). The slice was divided into two hemispheres along the midline, and each hemisphere was placed into an individual well of a custom slice incubation chamber [82], where it remained in oxygenated aCSF at 32–36°C until use. During recording, slices were transferred to a submerged recording chamber, where they were continuously perfused at 2 mL/minute with oxygenated aCSF with picrotoxin (100 μM) and maintained at 32–36°C. Voltage-clamp whole-cell recordings were obtained with borosilicate glass pipettes (2–5 MΩ) filled with the following solution (in mM): 130 CsMeSO_3_, 20 CsCl, 5 NaCl, 10 HEPES, 0.6 EGTA, 20 TEA, 4 MgATP, 0.3 Na_2_GTP, with a pH of 7.3 and osmolarity of 290 mOsmol. Cells were recorded only if the initial series resistance was ≤20 MΩ and were excluded from analysis if the series resistance (Rs) changed more than 20% during the recording period. Evoked EPSCs were recorded using a bipolar stimulating electrode located within the striatum and approximately 200 μm away, medial dorsal to the soma (along the fiber bundles). AMPAR- and NMDAR-mediated components were identified according to their distinct activation mechanisms and deactivation kinetics [83,84]. AMPAR-mediated EPSCs were recorded at −70 mV and measured as the peak response following the stimulus. NMDAR-mediated EPSCs were recorded at +40 mV and measured as the mean current over a 5-ms window, 50 ms after the stimulus. Mean EPSCs were an average of 10–15 sweeps obtained at 0.1 Hz.

For electrophysiological recordings from CINs, mice aged 5 weeks were anesthetized with isoflurane for preparation of striatal-containing brain slices. Slicing was done in bubbled ice-cold 95% O_2_/5% CO_2_–equilibrated solution containing (in mM) the following: 92 choline chloride, 2.5 KCl, 1.2 NaH_2_PO_4_, 30 NaHCO_3_, 20 HEPES, 25 glucose, 5 sodium ascorbate, 2 thiourea, 3 sodium pyruvate, 10 MgSO_4_, and 0.5 CaCl_2_. Coronal slices (280 μm) were prepared and transferred for 10 minutes to a warmed solution (34 °C) of identical composition before they were transferred at approximately 22 °C in 95% O_2_/5% CO_2_–equilibrated aCSF containing (in mM) the following: 92 sodium chloride, 2.5 KCl, 1.2 NaH_2_PO_4_, 30 NaHCO_3_, 20 HEPES, 25 glucose, 5 sodium ascorbate, 2 thiourea, 3 sodium pyruvate, 2 MgSO_4_, and 2 CaCl_2_. Recordings were performed with a Scientifica SliceScope system with aCSF containing the following (in mM): 126 NaCl, 2.5 KCl, 2 CaCl2, 2 MgCl2, 18 NaHCO3, 1.2 NaH2PO4, 10 glucose (flow rate of approximately 2  mL   m^–1^). CINs were identified based on large soma size, as well as by their characteristic tonic AP firing observed in the cell-attached mode. Pipettes (3–5 MΩ) were pulled from P-1000 (Sutter Instruments, CA) and filled with an intracellular solution containing the following (in mM): 119 K-MeSO4, 12 KCl, 1 MgCl2, 0.1 CaCl2, 10 HEPES, 1 EGTA, 0.4 Na-GTP, 2 Mg-ATP (280–300 mOsm, pH 7.3 adjusted with KOH). Spikes were evoked using current step injections (500-ms duration at 0.2 Hz, −180- to +300-pA range with increasing 30-pA steps). Input resistance was measured with a 120-pA hyperpolarizing step from the RMP. Acquisition was done using Clampex 10.5, MultiClamp 700B amplifier, and Digidata 1440A (Molecular Devices, CA). Data were analyzed with Clampfit 10.5.

### Data analysis

Statistical analyses were performed using Prism (GraphPad Software; La Jolla, CA). Data are presented throughout as the mean ± SEM. Student *t* test, one-way and two-way ANOVA, followed by Bonferroni’s post hoc test were used, as appropriate. The minimal level of significance was set at *P* < 0.05.

## Supporting information

S1 Fig Lack of NF1 in D1R-MSN or D2R-MSN on food self-administration. (A) Western blots and quantification of NF1 levels in the striatum of *Nf1*^*flx/flx*^*D1*^*Cre*^ and *Nf1*^*flx/flx*^ mice. *n* = 4 mice/group, Student *t* test. (B) Number of active lever presses across food and morphine self-administration paradigm for *Nf1*^*flx/flx*^ and *Nf1*^*flx/flx*^*D1*^*Cre*^ mice. *n* = 5–8 mice/group, two-way RM ANOVA. (C) Western blots and quantification of NF1 levels in the striatum of *Nf1*^*flx/flx*^*D2*^*Cre*^ and *Nf1*^*flx/flx*^ mice. *n* = 4 mice/group, Student *t* test. (D) Number of active lever presses across food and morphine self-administration paradigm for *Nf1*^*flx/flx*^ and *Nf1*^*flx/flx*^*D2*^*Cre*^ *mice*. *n* = 6–10 mice/group, two-way RM ANOVA. **P < 0.01, ***P < 0.001, **** *P* < 0.0001, data are represented as mean + SEM. Underlying data for this figure can be found in S1 Data. D1R, D1 dopamine receptor; D2R, D2 dopamine receptor; MSN, medium spiny neuron; NF1, neurofibromin 1; RM-ANOVA, repeated measures analysis of variance.
(TIF)

S2 Fig MSNs in the NAc lacking NF1 are resistant to morphine-induced reduction in excitability. (A) Representative traces of NAc MSN spiking activity at 250-pA injection level and (B) the mean number of APs generated for a given level of current injection in *Nf1*^*flx/flx*^ and *Nf1*^*flx/flx*^*Rgs9*^*Cre*^ mice following morphine administration. Two-way RM ANOVA. (C) Comparison of firing threshold (rheobase), (D) RMP, and (E) input resistance (Rin) for drug-naïve and morphine-treated *Nf1*^*flx/flx*^ and *Nf1*^*flx/flx*^*Rgs9*^*Cre*^ mice. *n* = 8–12 mice/group, two-way ANOVA. (F) Representative traces and (G) summarized data showing AMPAR/NMDAR current ratio (*n* = 4–9 mice/genotype). The AMPAR component was measured as the maximal response while neurons were held at −70 mV. The NMDAR component was measured as the average current between 50 and 55 ms (square) following the stimulation while the neurons were held at +40 mV. (H) Representative traces and (I) data showing paired-pulse ratio comparison. *P < 0.05, **P < 0.01, ****P < 0.0001. Data are represented as mean + SEM. Underlying data for this figure can be found in S1 Data. AMPAR, α-amino-3-hydroxy-5-methyl-4-isoxazolepropionic acid receptor; AP, action potential; MSN, medium spiny neuron; NAc, nucleus accumbens; NF1, neurofibromin 1; NMDAR, N-methyl-D-aspartate receptor; RM-ANOVA, repeated measures analysis of variance; RMP, resting membrane potential.
(TIF)

S3 Fig Morphine increases NF1 protein levels in the striatum. (A) Representative western blots and quantification showing effects of morphine administration (10 mg/kg) on NF1. *n* = 5–6, Student *t* test, (B) representative western blots and quantification showing effects of morphine administration (20 mg/kg) on NF1, p120GAP, SynGAP, sos1, RasGRP1, and RasGRF2 levels. *n* = 4, Student *t* test, *P < 0.05, ***P < 0.01. Data are represented as mean + SEM. Underlying data for this figure can be found in S1 Data. NF1, neurofibromin 1
(TIF)

S4 Fig Excitability of CIN in the NAc of *Nf1^flx/flx^D2^Cre^* mice. (A) Image of CIN. (B) Representative traces of CIN spiking activity and (C) the mean number of APs generated for a given level of current injection in *Nf1*^*flx/flx*^ and *Nf1*^*flx/flx*^*D2*^*Cre*^. (D) Comparison of RMP and (E) input resistance (Rin) of *Nf1*^*flx/flx*^ and *Nf1*^*flx/flx*^*D2*^*Cre*^ mice. *n* = 12 mice/group. Underlying data for this figure can be found in S1 Data. AP, action potential; CIN, cholinergic interneuron; NAc, nucleus accumbens; RMP, resting membrane potential.
(TIF)

S5 Fig Elimination of striatal NF1 on motor function. (A) Daily performance of *Nf1*^*flx/flx*^ and *Nf1*^*flx/flx*^*Rgs9*^*cre*^ mice on the accelerating rotarod over 10 days (4 trials/day). *n* = 8–13 mice/group, two-way RM ANOVA. Underlying data for this figure can be found in S1 Data. NF1, neurofibromin 1; RM-ANOVA, repeated measures analysis of variance.
(TIF)

S6 Fig Elimination of striatal NF1 on motor function. (A) Grip strength (*n* = 6 mice/group) and (B) wire hang for *Nf1*^*flx/flx*^ and *Nf1*^*flx/flx*^*D2*^*cre*^ mice. *n* = 9 mice/group, Student *t* test. (C) Daily performance of *Nf1*^*flx/flx*^ and *Nf1*^*flx/flx*^*D2*^*cre*^ mice on the accelerating rotarod over 9 days (four trials/day). Data are represented as mean + SEM. Underlying data for this figure can be found in S1 Data. NF1, neurofibromin 1
(TIF)

S7 Fig AC5, Gαo, and Gαolf levels in the striatum of conditional NF1 knockout mice. (A) Representative western blots and quantification of Gαolf and Gαo in *Nf1*^*flx/flx*^*Rgs9*^*cre*^ mice. *n* = 5–8 mice/genotype. Representative western blots and quantification of AC5, Gαo, and Gαolf in the striatum, (B) *Nf1*^*flx/flx*^*D1*^*cre*^ mice (*n* = 5–7 mice/genotype) and (C) *Nf1*^*flx/flx*^*D2*^*cre*^ mice (*n* = 5–7 mice/genotype). Student *t* test, *P < 0.01. Data are represented as mean + SEM. Underlying data for this figure can be found in S1 Data. AC5, adenylyl cyclase type 5
(TIF)

S8 Fig Effects of DREADDs or CNO administration on rotarod task. Effects of bilateral injection of Gs-DREADD DIO-rM3D_s_ or control DREADD DIO-mcherry in the striatum of *Nf1*^*flx/flx*^*D2*^*cre*^ mice on (A) performance (two-way RM ANOVA) and (B) learning rate (Student *t* test) in the accelerating rotarod task. *n* = 4. Effects of bilateral injection of Gs-DREADD DIO-rM3D_s_ or control DREADD DIO-mcherry in the striatum of *Nf1*^*flx/flx*^*D2*^*cre*^ mice on (C) total distance (Student *t* test) and (D) time course in the open field assay (two-way RM ANOVA). *n* = 5–6 mice/group, effects of CNO on (E) performance and (F) learning rate in the accelerating rotarod task. *n* = 6 mice/group. Data are represented as mean + SEM. Underlying data for this figure can be found in S1 Data. DREADD, Designer Receptors Exclusively Activated by Designer Drugs; CNO, Clozapine-N-oxide; DIO, double-floxed inverted open reading frame; RM-ANOVA, repeated measures analysis of variance.
(TIF)

S9 Fig Effects of rolipram on NF1-deficit mice in the rotarod task. (A) Probability of reaching maximal speed over the 3 days of training on the accelerating rotarod for *Nf1*^*flx/flx*^*Rgs9*^*cre*^ mice treated with rolipram (Rol.) or vehicle (Veh.). *n* = 7–15 mice/group. (B) Performance (two-way RM ANOVA) and (C) learning rate of *Nf1*^*flx/flx*^*D2*^*cre*^ mice treated with rolipram or vehicle (two-way ANOVA). *n* = 9–10 mice/group. Data are represented as mean + SEM. Underlying data for this figure can be found in S1 Data. NF1, neurofibromin 1; RM-ANOVA, repeated measures analysis of variance.
(TIF)

S1 Data Numerical data used in Figs 1–7 and S1–S9 Figs. (XLSX)

## Abbreviations

aCSF: artificial cerebrospinal fluid
AC5: adenylyl cyclase type 5
AMPAR: α-amino-3-hydroxy-5-methyl-4-isoxazolepropionic acid receptor
AP: action potential
BAC: bacterial artificial chromosome
CAMPER: cAMP encoded reporter
CIN: cholinergic interneuron
CNO: clozapine-N-oxide
CPP: conditioned place preference
DIO: double-floxed inverted open reading frame
DREADD: Designer Receptors Exclusively Activated by Designer Drugs
D1R: D1 dopamine receptor
D2R: D2 dopamine receptor
ERK: extracellular signal regulated kinase
FRET: Forster Resonance Transfer
FR1: fixed-ratio 1
GFP: green fluorescent protein
GPCR: G protein coupled receptor
GSK-3: glycogen synthase kinase 3
MAPK: mitogen-activated protein kinase
MOR: Mu opioid receptor
MSN: medium spiny neuron
mTOR: mammalian target of rapamycin
NAc: nucleus accumbens
NF1: neurofibromin 1
NMDAR: N-methyl-D-aspartate receptor
RasGAP: Ras-specific GTPase activating protein
RMP: resting membrane potential
SNc: substantia nigra pars compacta
TBS: Tris-buffered saline
