# Supplementary figures and images for: NF1-cAMP signaling dissociates cell type–specific contributions of striatal medium spiny neurons to reward valuation and motor control
Source: PLoS Biol. 2019 Oct 10;17(10):e3000477. doi: 10.1371/journal.pbio.3000477 (PMC6805008; doi:10.1371/journal.pbio.3000477)

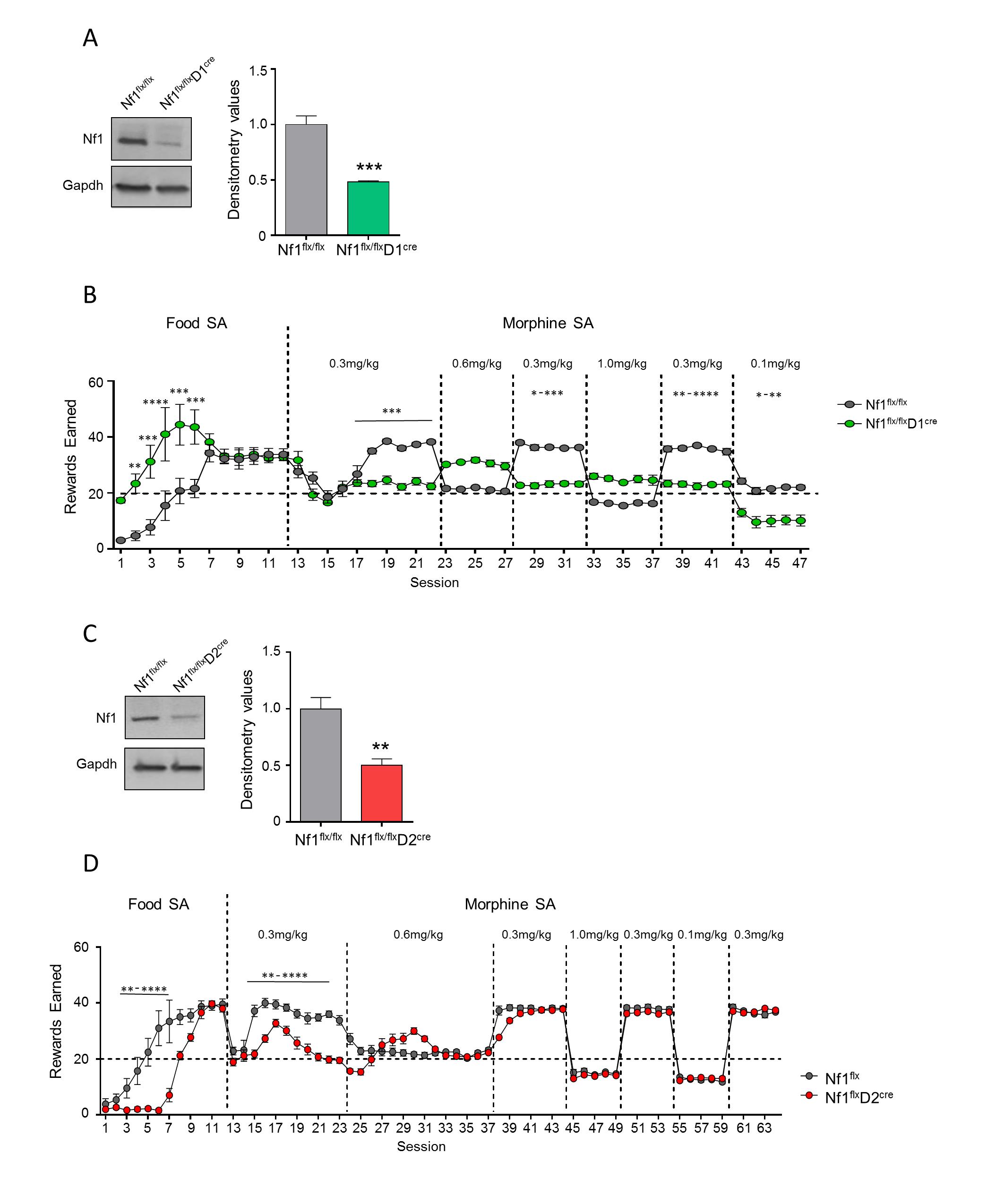

Supplement: S1 Fig — (A) Western blots and quantification of NF1 levels in the striatum of Nf1flx/flxD1Cre and Nf1flx/flx mice. n = 4 mice/group, Student t test. (B) Number of active lever presses across food and morphine self-administration paradigm for Nf1flx/flx and Nf1flx/flxD1Cre mice. n = 5–8 mice/group, two-way RM ANOVA. (C) Western blots and quantification of NF1 levels in the striatum of Nf1flx/flxD2Cre and Nf1flx/flx mice. n = 4 mice/group, Student t test. (D) Number of active lever presses across food and morphine self-administration paradigm for Nf1flx/flx and Nf1flx/flxD2Cre mice. n = 6–10 mice/group, two-way RM ANOVA. **P < 0.01, ***P < 0.001, **** P < 0.0001, data are represented as mean + SEM. Underlying data for this figure can be found in S1 Data. D1R, D1 dopamine receptor; D2R, D2 dopamine receptor; MSN, medium spiny neuron; NF1, neurofibromin 1; RM-ANOVA, repeated measures analysis of variance. (TIF) [file pbio.3000477.s001.tif]

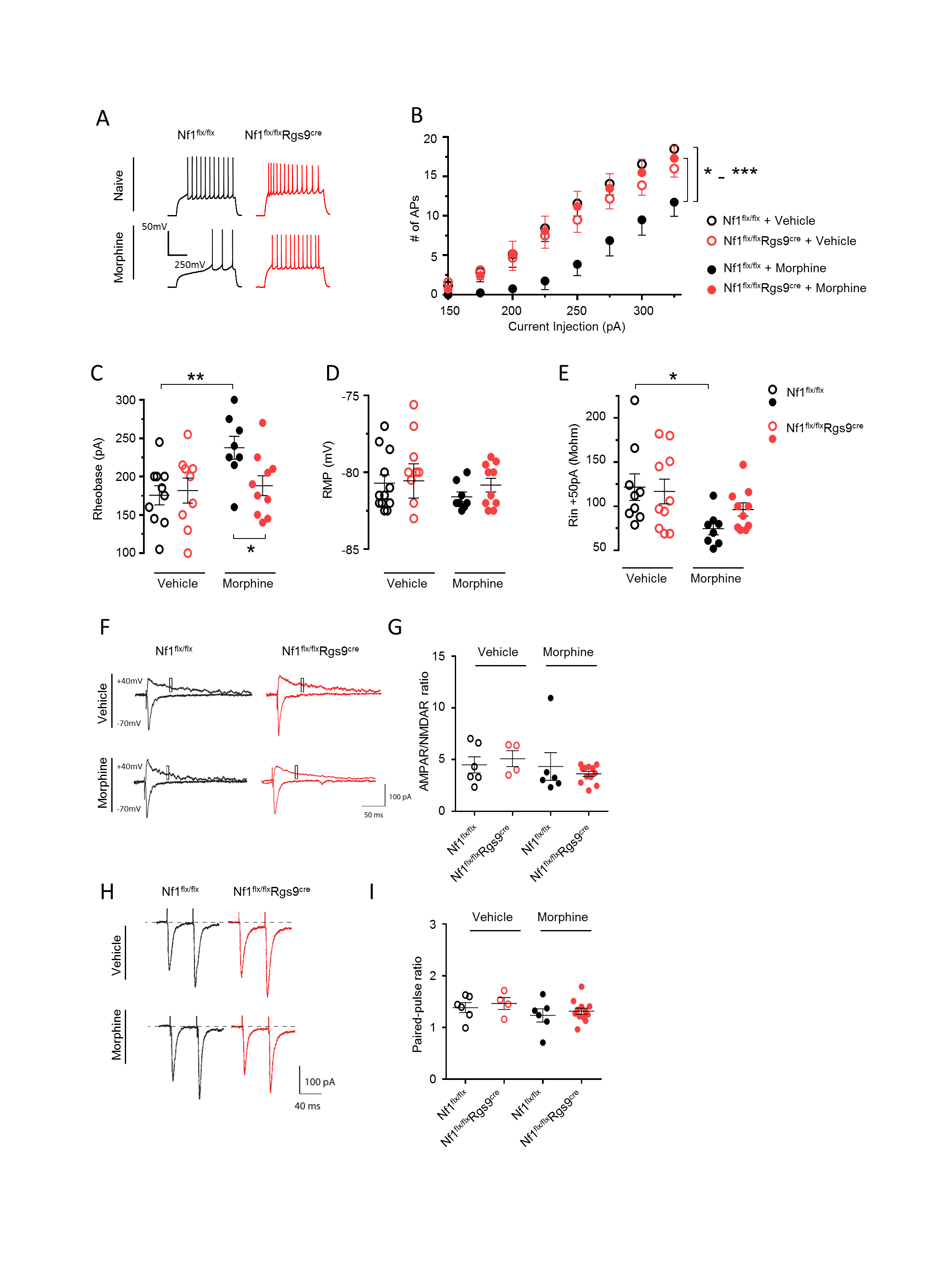

Supplement: S2 Fig — (A) Representative traces of NAc MSN spiking activity at 250-pA injection level and (B) the mean number of APs generated for a given level of current injection in Nf1flx/flx and Nf1flx/flxRgs9Cre mice following morphine administration. Two-way RM ANOVA. (C) Comparison of firing threshold (rheobase), (D) RMP, and (E) input resistance (Rin) for drug-naïve and morphine-treated Nf1flx/flx and Nf1flx/flxRgs9Cre mice. n = 8–12 mice/group, two-way ANOVA. (F) Representative traces and (G) summarized data showing AMPAR/NMDAR current ratio (n = 4–9 mice/genotype). The AMPAR component was measured as the maximal response while neurons were held at −70 mV. The NMDAR component was measured as the average current between 50 and 55 ms (square) following the stimulation while the neurons were held at +40 mV. (H) Representative traces and (I) data showing paired-pulse ratio comparison. *P < 0.05, **P < 0.01, ****P < 0.0001. Data are represented as mean + SEM. Underlying data for this figure can be found in S1 Data. AMPAR, α-amino-3-hydroxy-5-methyl-4-isoxazolepropionic acid receptor; AP, action potential; MSN, medium spiny neuron; NAc, nucleus accumbens; NF1, neurofibromin 1; NMDAR, N-methyl-D-aspartate receptor; RM-ANOVA, repeated measures analysis of variance; RMP, resting membrane potential. (TIF) [file pbio.3000477.s002.tif]

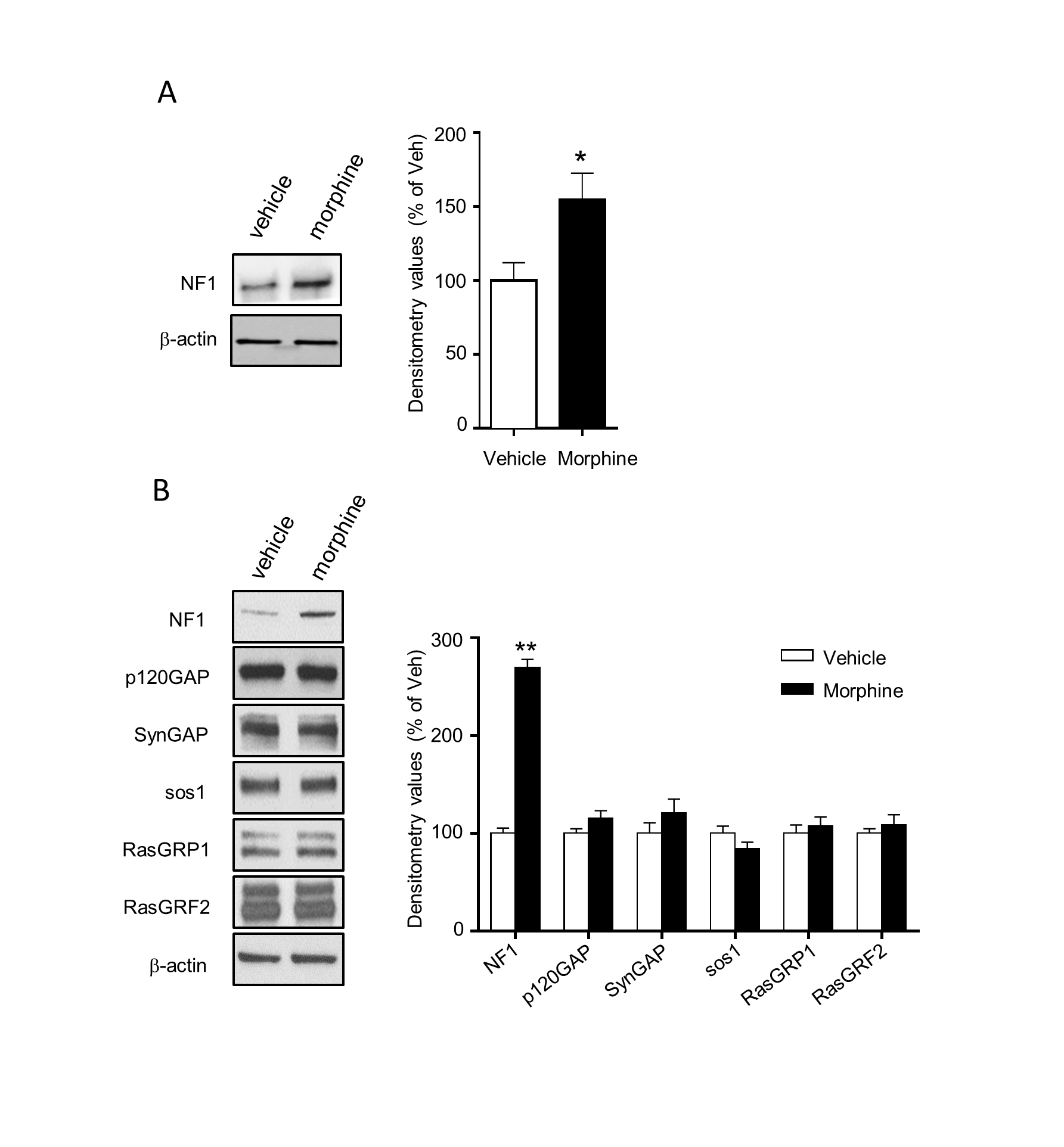

Supplement: S3 Fig — (A) Representative western blots and quantification showing effects of morphine administration (10 mg/kg) on NF1. n = 5–6, Student t test, (B) representative western blots and quantification showing effects of morphine administration (20 mg/kg) on NF1, p120GAP, SynGAP, sos1, RasGRP1, and RasGRF2 levels. n = 4, Student t test, *P < 0.05, ***P < 0.01. Data are represented as mean + SEM. Underlying data for this figure can be found in S1 Data. NF1, neurofibromin 1 (TIF) [file pbio.3000477.s003.tif]

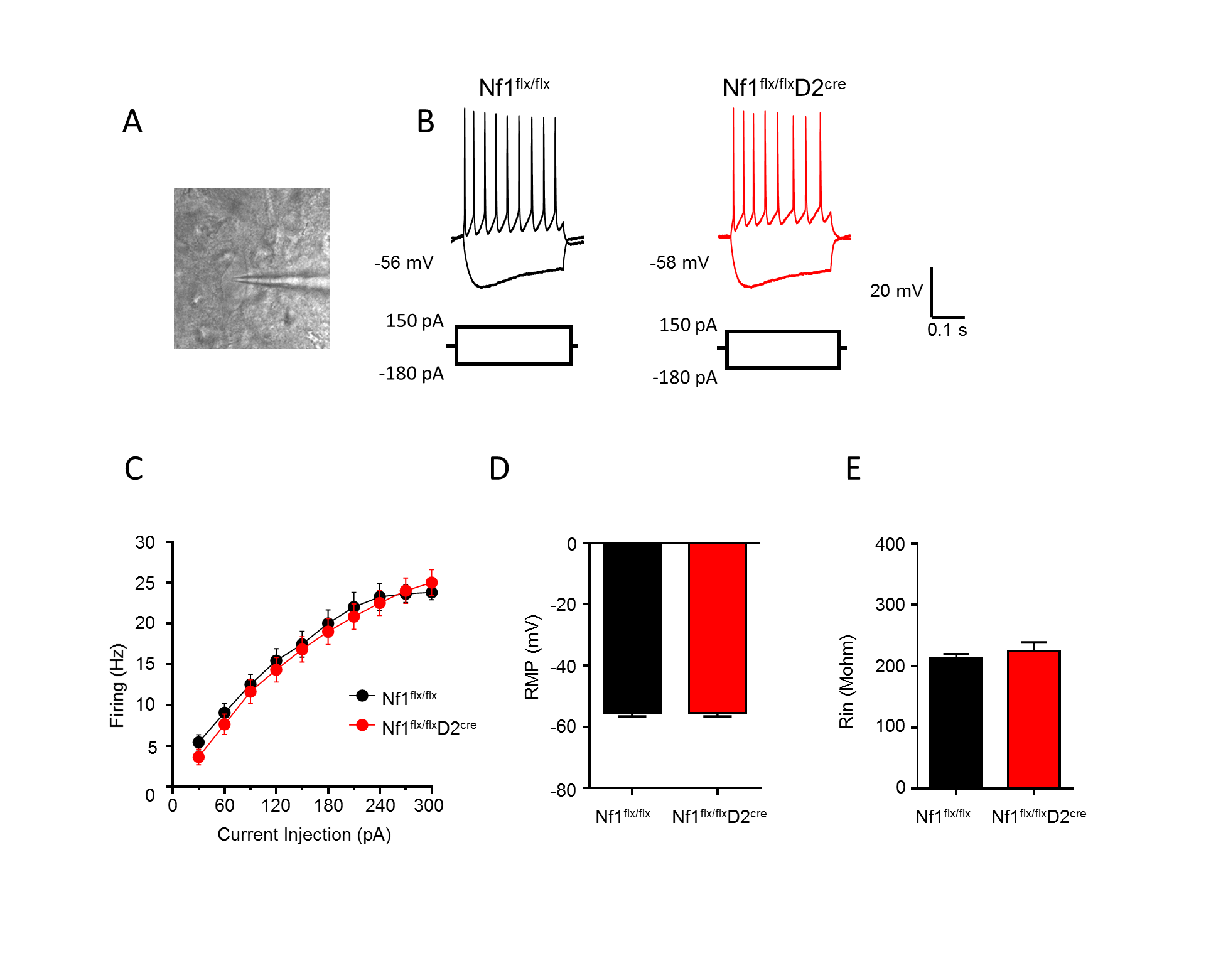

Supplement: S4 Fig — (A) Image of CIN. (B) Representative traces of CIN spiking activity and (C) the mean number of APs generated for a given level of current injection in Nf1flx/flx and Nf1flx/flxD2Cre. (D) Comparison of RMP and (E) input resistance (Rin) of Nf1flx/flx and Nf1flx/flxD2Cre mice. n = 12 mice/group. Underlying data for this figure can be found in S1 Data. AP, action potential; CIN, cholinergic interneuron; NAc, nucleus accumbens; RMP, resting membrane potential. (TIF) [file pbio.3000477.s004.tif]

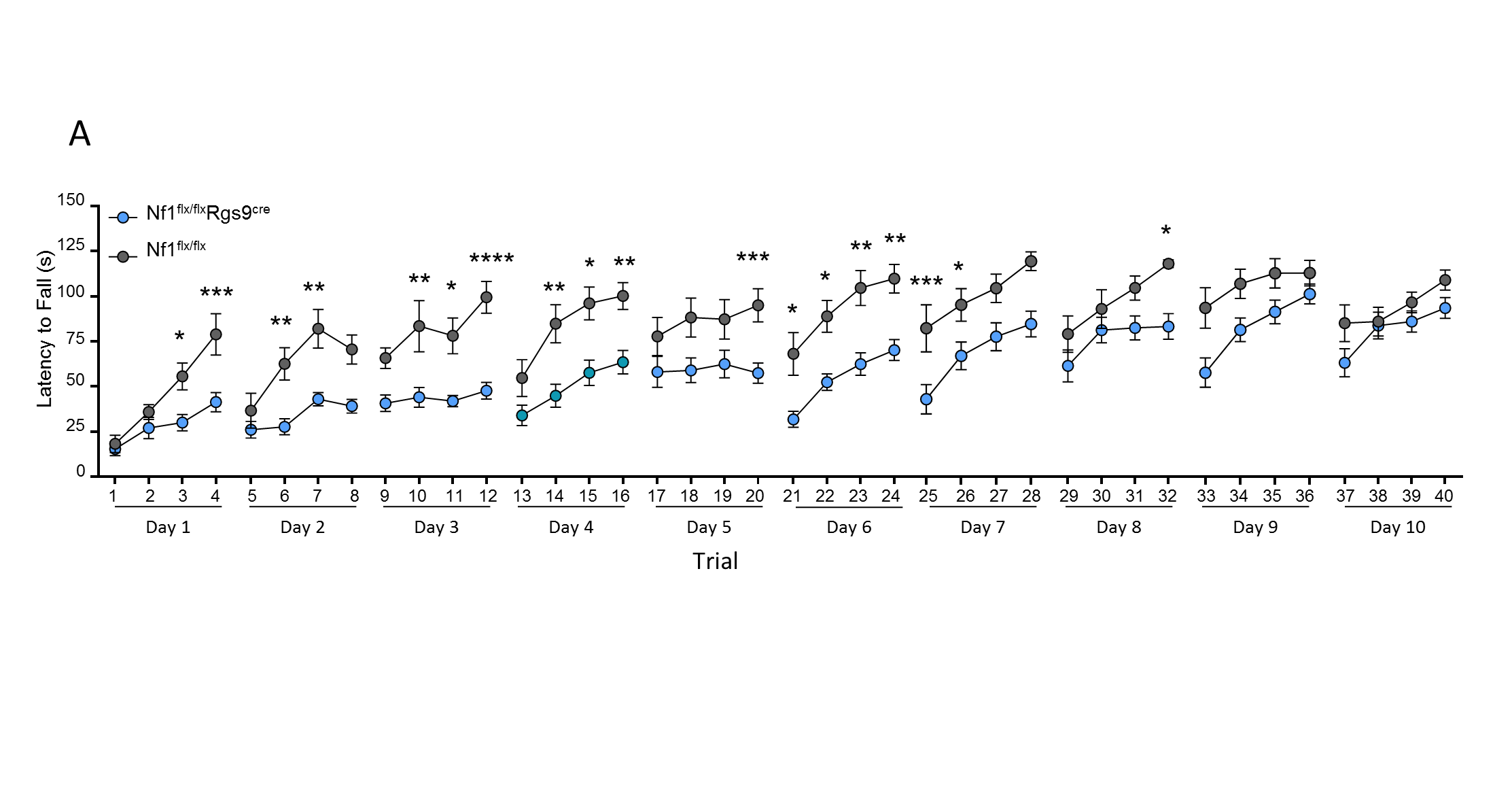

Supplement: S5 Fig — (A) Daily performance of Nf1flx/flx and Nf1flx/flxRgs9cre mice on the accelerating rotarod over 10 days (4 trials/day). n = 8–13 mice/group, two-way RM ANOVA. Underlying data for this figure can be found in S1 Data. NF1, neurofibromin 1; RM-ANOVA, repeated measures analysis of variance. (TIF) [file pbio.3000477.s005.tif]

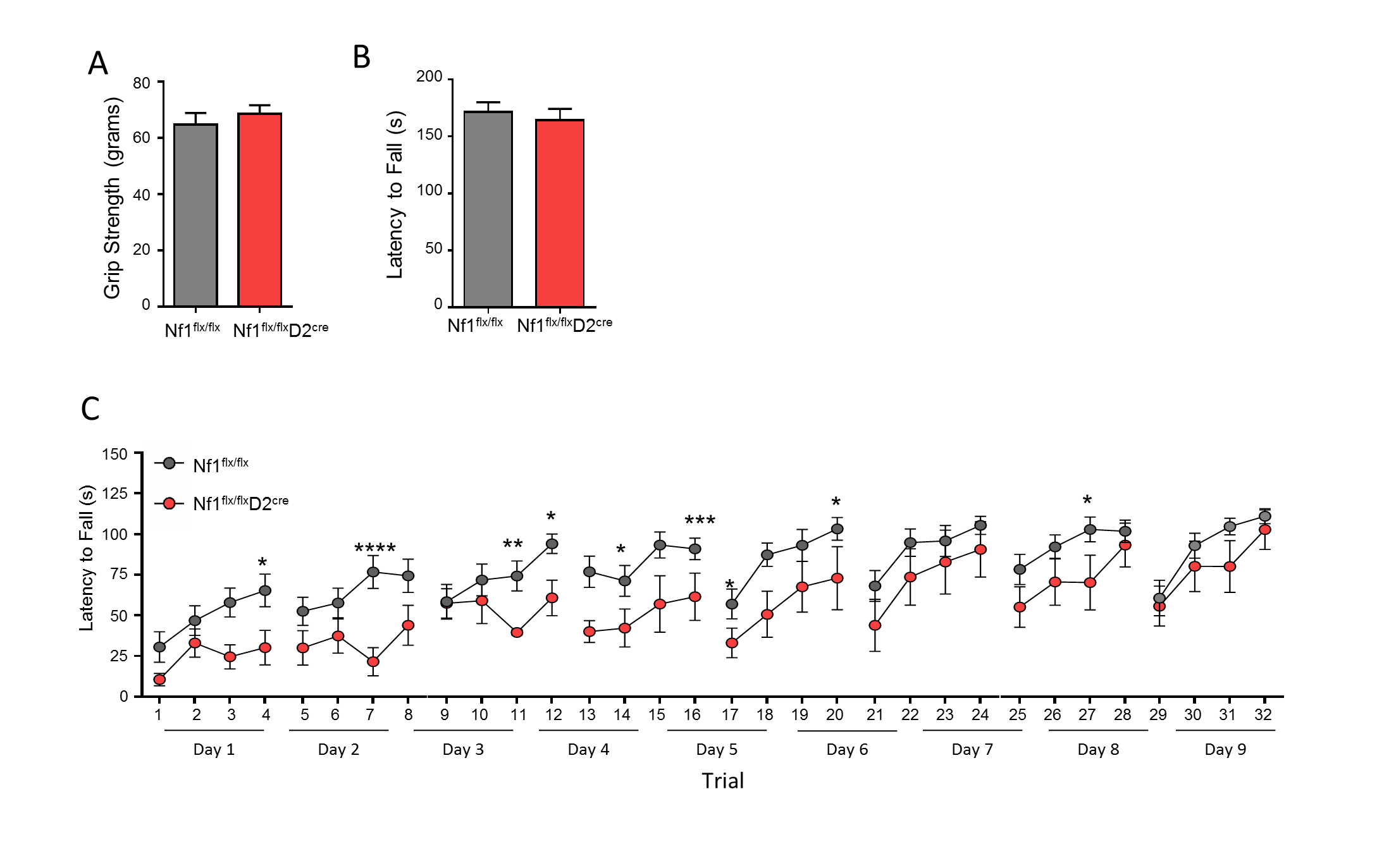

Supplement: S6 Fig — (A) Grip strength (n = 6 mice/group) and (B) wire hang for Nf1flx/flx and Nf1flx/flxD2cre mice. n = 9 mice/group, Student t test. (C) Daily performance of Nf1flx/flx and Nf1flx/flxD2cre mice on the accelerating rotarod over 9 days (four trials/day). Data are represented as mean + SEM. Underlying data for this figure can be found in S1 Data. NF1, neurofibromin 1 (TIF) [file pbio.3000477.s006.tif]

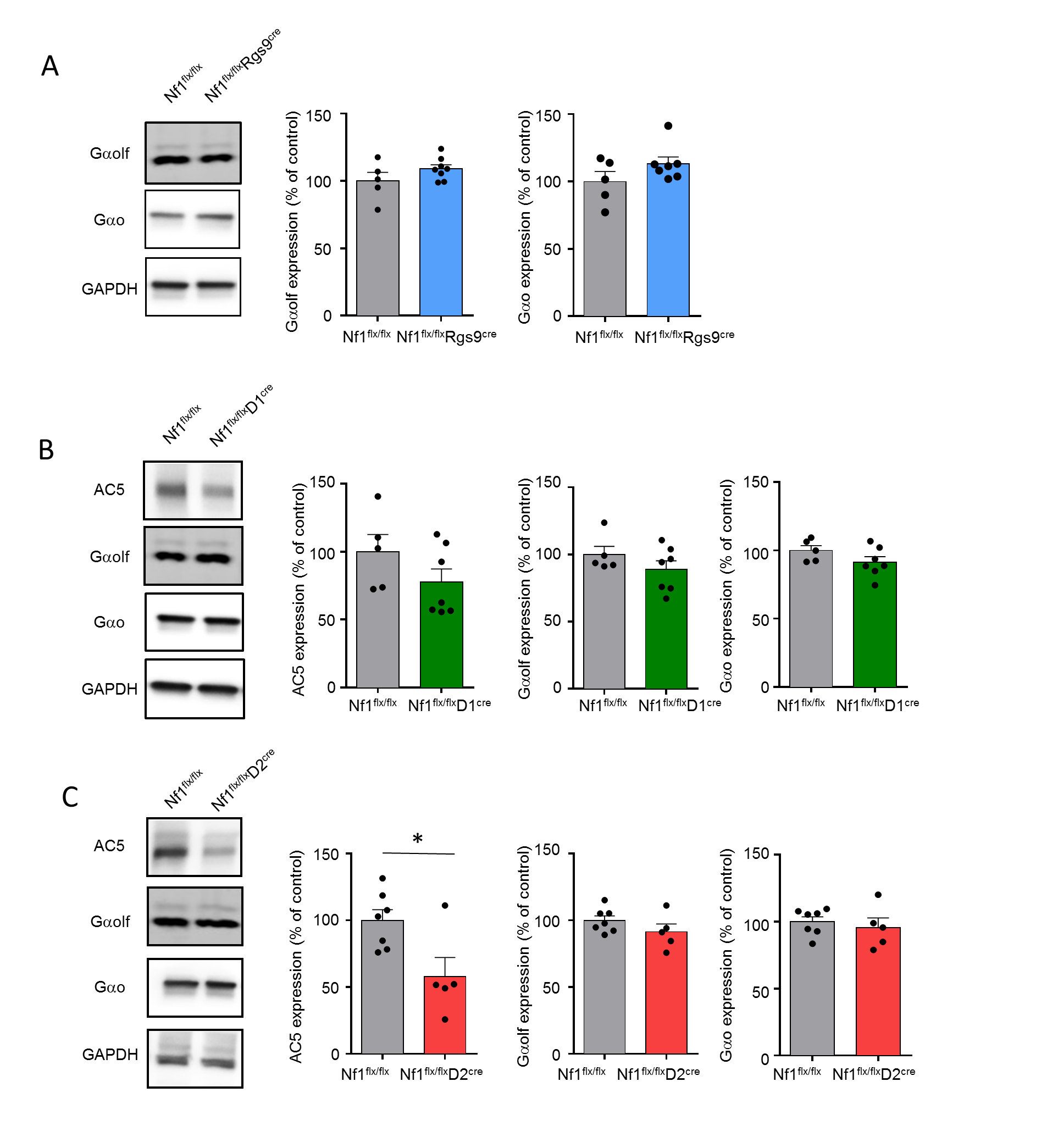

Supplement: S7 Fig — (A) Representative western blots and quantification of Gαolf and Gαo in Nf1flx/flxRgs9cre mice. n = 5–8 mice/genotype. Representative western blots and quantification of AC5, Gαo, and Gαolf in the striatum, (B) Nf1flx/flxD1cre mice (n = 5–7 mice/genotype) and (C) Nf1flx/flxD2cre mice (n = 5–7 mice/genotype). Student t test, *P < 0.01. Data are represented as mean + SEM. Underlying data for this figure can be found in S1 Data. AC5, adenylyl cyclase type 5 (TIF) [file pbio.3000477.s007.tif]

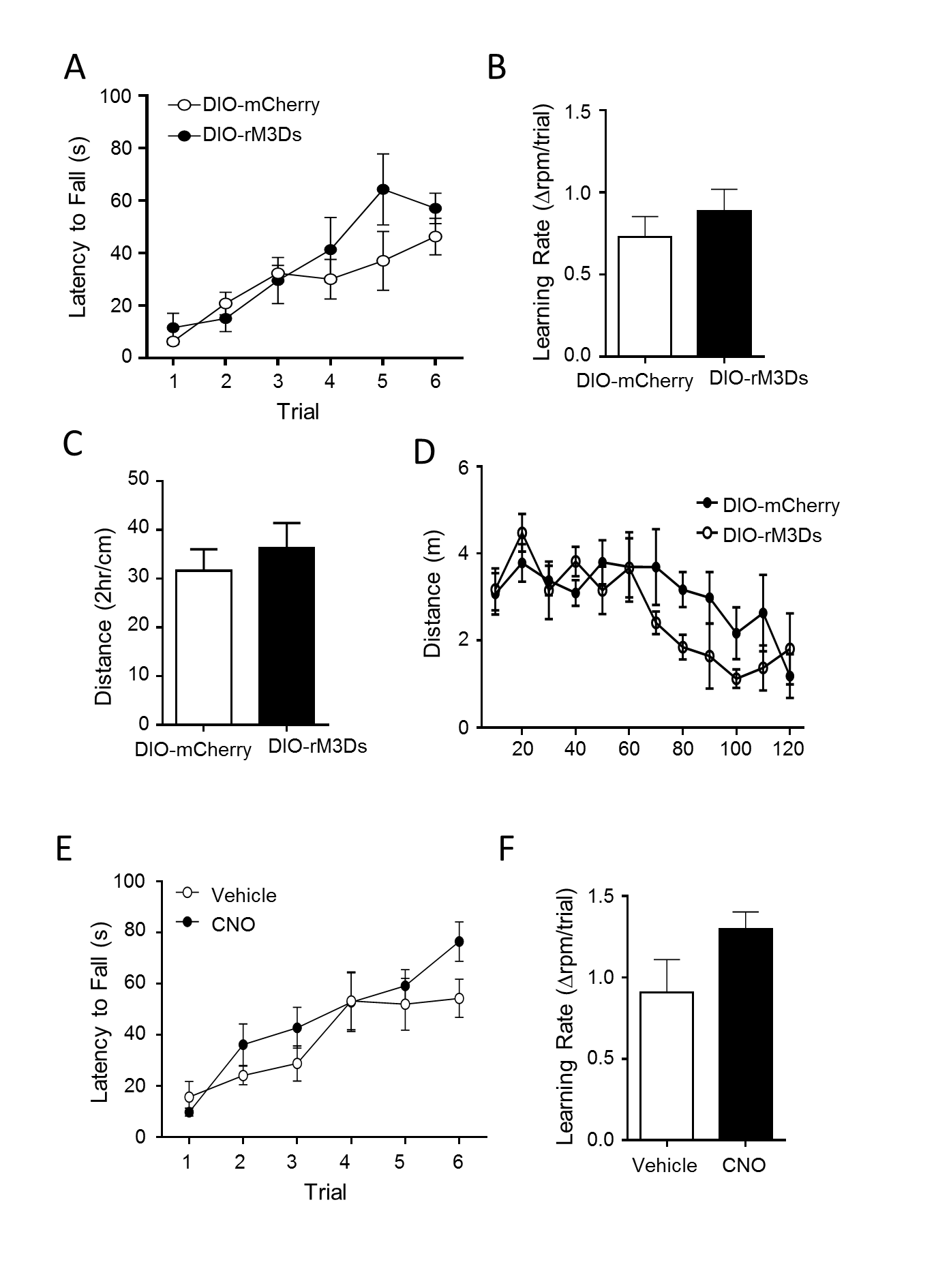

Supplement: S8 Fig — Effects of bilateral injection of Gs-DREADD DIO-rM3Ds or control DREADD DIO-mcherry in the striatum of Nf1flx/flxD2cre mice on (A) performance (two-way RM ANOVA) and (B) learning rate (Student t test) in the accelerating rotarod task. n = 4. Effects of bilateral injection of Gs-DREADD DIO-rM3Ds or control DREADD DIO-mcherry in the striatum of Nf1flx/flxD2cre mice on (C) total distance (Student t test) and (D) time course in the open field assay (two-way RM ANOVA). n = 5–6 mice/group, effects of CNO on (E) performance and (F) learning rate in the accelerating rotarod task. n = 6 mice/group. Data are represented as mean + SEM. Underlying data for this figure can be found in S1 Data. DREADD, Designer Receptors Exclusively Activated by Designer Drugs; CNO, Clozapine-N-oxide; DIO, double-floxed inverted open reading frame; RM-ANOVA, repeated measures analysis of variance. (TIF) [file pbio.3000477.s008.tif]

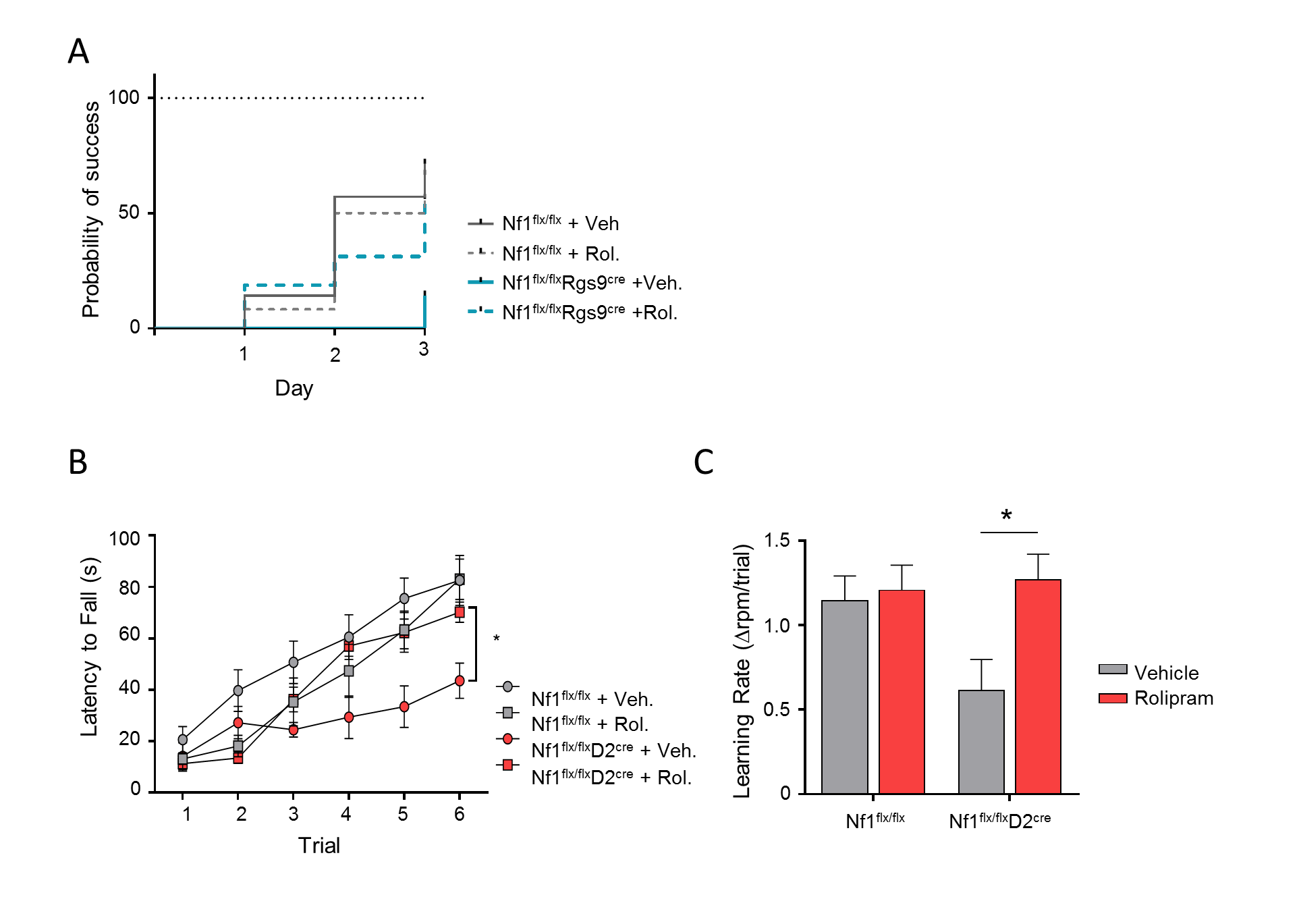

Supplement: S9 Fig — (A) Probability of reaching maximal speed over the 3 days of training on the accelerating rotarod for Nf1flx/flxRgs9cre mice treated with rolipram (Rol.) or vehicle (Veh.). n = 7–15 mice/group. (B) Performance (two-way RM ANOVA) and (C) learning rate of Nf1flx/flxD2cre mice treated with rolipram or vehicle (two-way ANOVA). n = 9–10 mice/group. Data are represented as mean + SEM. Underlying data for this figure can be found in S1 Data. NF1, neurofibromin 1; RM-ANOVA, repeated measures analysis of variance. (TIF) [file pbio.3000477.s009.tif]
